# Lipin1 as a therapeutic target for respiratory insufficiency of duchenne muscular dystrophy

**DOI:** 10.3389/fphys.2024.1477976

**Published:** 2024-11-12

**Authors:** Alexandra Brown, Brooklyn Morris, John Karanja Kamau, Ryan J. Rakoczy, Brian N. Finck, Christopher N. Wyatt, Hongmei Ren

**Affiliations:** ^1^ Department of Biochemistry and Molecular Biology, Wright State University, Dayton, OH, United States; ^2^ Department of Neuroscience, Cell Biology and Physiology, Wright State University, Dayton, OH, United States; ^3^ Division of Nutritional Science and Obesity Medicine, Washington University School of Medicine in St. Louis, St. Louis, MO, United States

**Keywords:** lipin1, DMD, muscular dystrophy, dystrophin, diaphragm, skeletal muscle, therapeutic target, membrane integrity

## Abstract

In Duchenne muscular dystrophy (DMD), diaphragm muscle dysfunction results in respiratory insufficiency which is a leading cause of death in patients. Mutations to the dystrophin gene result in myocyte membrane instability, contributing to the structural deterioration of the diaphragm muscle tissues. With previous works suggesting the importance of lipin1 for maintaining skeletal muscle membrane integrity, we explored the roles of lipin1 in the dystrophic diaphragm. We found that the protein expression levels of lipin1 were reduced by 60% in the dystrophic diaphragm. While further knockdown of lipin1 in the dystrophic diaphragm leads to increased necroptosis, restoration of lipin1 in the dystrophic diaphragm results in reduced inflammation and fibrosis, decreased myofiber death, and improved respiratory function. Our results demonstrated that lipin1 restoration improved respiratory function by enhancing membrane integrity and suggested that lipin1 could be a potential therapeutic target for preventing respiratory insufficiency and respiratory failure in DMD. Continued investigation is required to better understand the mechanisms behind these findings, and to determine the role of lipin1 in maintaining muscle membrane stability.

## Background

Duchenne muscular dystrophy (DMD) is an X-linked recessive disorder that is characterized by severe and progressive muscle weakness and muscle wasting including respiratory muscles (Duan et al., 2021). This disease is caused by a mutation in the largest known human gene which encodes the protein, dystrophin. Dystrophin is a cytoskeletal protein connecting the inner cytoskeleton to the extracellular matrix and serves as a molecular shock absorber to maintain sarcolemmal stability and protect the muscles from injury (Le et al., 2018). Dystrophin deficiency leads to impairment of skeletal muscle sarcolemmal membrane integrity, including the diaphragm, rendering the membrane susceptible to mechanical damage during muscle contraction, leading to immune cell infiltration, necrosis, and fibrosis (Bez Batti Angulski et al., 2023). These histopathological abnormalities of the respiratory muscle have been shown to lead to reduced lung and chest wall compliance, decreased ventilation, and alveolar hypoventilation in many boys with DMD (Smith et al., 1989; Barbe et al., 1994). Patients with DMD have reduced ventilatory capacity beginning at a young age and will decline with age (Burns et al., 2019; Khirani et al., 2014). Respiratory insufficiency is ubiquitous in patients at older age and respiratory failure is one of the leading causes of morbidity and mortality in muscular dystrophy, with a mean life expectancy between 14.4 and 27.0 years unless artificially supported by mechanical ventilation (Pennati et al., 2021; Landfeldt et al., 2020).

A primary strategy to treat DMD would be to reverse the instability of muscle membranes by increasing dystrophin levels. However, a major obstacle to that approach is that the dystrophin gene is too large to be packaged into current gene therapy vectors. While Elevidys micro-dystrophin gene therapy has recently been approved by FDA, these highly truncated forms of dystrophin do not offer full protection (Elangkovan and Dickson, 2021). Moreover, exon-skipping therapies including Amondys 45, Exondys 51, Viltepso, and Vyondys 53 are only effective for some dystrophin mutations (Eser and Topaloglu, 2022). Therefore, it is critical to identify new molecules that are important for ameliorating dystrophic phenotypes and restoring diaphragm function in DMD.

Lipin1 is a phosphatidic acid (PA) phosphatase (PAP) that catalyzes the conversion of PA into diacylglycerol (DAG), which is a critical step in the synthesis of glycerophospholipids, the major structural lipid component of cellular membranes (Csaki and Reue, 2010). Lipin1 accounts for most of the PAP activity in skeletal muscles (Reue and Dwyer, 2009). We recently discovered that lipin1 is critical for maintaining membrane integrity, and plays complementary roles in myofiber stability and muscle function in dystrophic skeletal muscles (Jama et al., 2023). Lipin1 deficiency in skeletal muscle led to membrane damage indicated by increased Evans blue dye (EBD) leakage (Ramani et al., 2020; Schweitzer et al., 2019). Increased membrane permeability was associated with elevated cell death markers and inflammation. This is consistent with our cell culture study, lipin1 deficiency in differentiated primary myoblasts leads to increased membrane permeability and upregulated necroptotic markers (Jama et al., 2023). In contrast, increasing lipin1 expression levels suppressed the necroptotic markers (Jama et al., 2023).

In this study, we aimed to explore the role of lipin1 in dystrophic pathogenesis and respiratory dysfunction through loss-of-function and gain-of-function studies in the *mdx* mouse diaphragm. We assessed the effects of knocking out lipin1 in dystrophic diaphragm histology and function using *dystrophin:lipin1-DKO* mice, and the effects of restoring lipin1 in dystrophic diaphragm using a unique muscle-specific transgenic mouse model, *mdx:lipin1* ^*Tg/0*^ *,* in which lipin1 is selectively restored in the muscles of *mdx* mice. We found that *DKO* mice had increased dystrophic disease severity, whereas increasing lipin1 expression levels led to decreased muscle fiber degeneration, suppressed inflammation, reduced fibrosis, strengthened membrane integrity, and improved respiratory function.

## Methods

### Animals

Skeletal muscle-specific lipin1 deficient (*lipin1* ^*Myf5cKO*^) mice were generated by crossing lipin1^fl/fl^ with mice expressing Cre recombinase driven by Myf5 promoter as described in our previous study (Jama et al., 2024). C57BL/10ScSnJ (B10, #000476) *WT* and *mdx* (#001801) mice were originally purchased from Jackson Laboratories (Bar Harbor, ME, United States). *Dystrophin/lipin1-DKO* mice were generated by crossing *lipin1* ^*Myf5cKO*^ with *mdx* mice as described in our previous study (Jama et al., 2023). Hemizygous *mdx:lipin1* ^*Tg/0*^ mice were generated by crossing *mdx* mice with muscle-specific lipin1 transgenic mice generated by crossing Rosa26-Stop-Lipin1 knockin (Rosa26-lipin1^KI^) mice with mice carrying the Cre recombinase driven by the muscle creatine kinase (MCK) gene promoter (Jama et al., 2024). Because DMD occurs primarily in males, male mice at 6–7 months of age were used in the present study. These mice had free access to drinking water and regular chow unless otherwise noted. All animal experiments were performed in accordance with the relevant guidelines and regulations approved by the Animal Care and Use Committee of Wright State University and approval was obtained for all experiments performed in the present study.

### Western blotting

As described in our previous studies (Jiang et al., 2015; Alshudukhi et al., 2018), diaphragm muscle tissues were homogenized using RIPA buffer containing 10 mM Tris-HCl (pH 8.0), 30 mM NaCl, 1 mM EDTA, and 1% Nonidet P-40, supplemented with proteinase inhibitors and phosphatase inhibitors before use. Protein concentration was determined for each sample by BCA assay to ensure that equal amounts of protein were used across all samples. The samples were boiled for 5 min with 4x loading dye and separated by 7.5%–15% SDS-PAGE. Proteins were transferred to polyvinylidene difluoride membranes (Millipore) using a Mini Trans-Blot Cell System (Bio-Rad). The membrane was blocked with 5% nonfat milk (9999; Cell Signaling Technologies) for 1 h, and incubated with the primary antibodies in 5% BSA (BP9704; Thermo Fisher Scientific) in TBST overnight at 4°C. After probing with secondary antibodies for 1 h at room temperature, protein bands were detected by using Amersham Imager 600 (GE Healthcare Life Sciences). GAPDH (Cell Signaling Technologies, dilution 1:5000, catalog 2118) antibody was used as a loading control. The primary antibodies from Cell Signaling Technology were used, at dilution 1:1000, include lipin1 (catalog 4906), RIPK3 (catalog 95702), RIPK1 (catalog 3493), MLKL (catalog 37705), Cleaved Caspase-3 (catalog 9664), SMAD2/3 (catalog 8685), pSMAD2 serine 465/567 (catalog 3108), NF-κB (catalog 8242), pNF-κB serine 468 (catalog 3039), pNF-κB serine 536 (catalog 3033), Bax (catalog 2772), and Bak (catalog 12105). The NIH ImageJ software was used to quantify all western blots by densitometry. The values obtained were normalized to the loading control.

### Evans blue dye (EBD) assay

Mice were injected with EBD (10 mg/mL stock in sterile saline, 0.1 mL/10 g body weight) by i.p. and euthanized 24 h later. The skeletal muscles were dissected and snap-frozen in isopentane-cooled optimal cutting temperature (OCT) embedding media (Tissue-Tek, Sakura-Americas). Frozen OCT blocks were cryo-sectioned at 10 μm thickness and stained with laminin antibody before being analyzed by fluorescence microscopy.

### Immunofluorescence, microscopy, and image processing

The diaphragm muscles were frozen and sectioned at 10 µm using a cryostat machine. Slides were stored at −20°C. For staining, muscle sections were air dried for 1 h at room temperature. The muscle sections were then hydrated with PBST, followed by blocking with 5% goat serum in PBST. To detect macrophage distribution, sections were incubated with antibodies against CD86 (Abcam, catalog ab239075, dilution 1:100), CD206 (Cell Signaling Technology, catalog 24595, dilution 1:1000), or laminin (Abcam, catalog ab11575, dilution 1:500) for 1 h at 37°C, and subsequently with an Alexa Fluor 488-conjugated secondary antibody (Thermo Fisher Scientific, catalog A-21411, dilution 1:250) for 1 h in the dark at room temperature. Images were obtained using an inverted microscope (Olympus, IX70) equipped with a DFC7000T camera (Leica Microsystems, Wetzlar, Germany). Indicated images were quantified by CellProfiler software (Brown et al., 2023).

### Respiratory function analysis

Respiratory function assay was performed using whole-body plethysmography (FinePointe; Buxco/DSI) in conscious, unrestrained animals. Pressure sensor calibration was achieved via direct injection of a 5 mL bolus of air into the experimental chamber and was automated by computer software. Animals were acclimatized to the plethysmography chamber (BUXCO Europe Ltd.) for 3 days before experiments. On the day of the experiments, animals were introduced into plethysmography chambers and allowed a 20-min acclimation period with constant flow (200 mL/min) of compressed air to prevent the buildup of CO_2_ and depletion of O_2_. Following the acclimation period, respiratory parameters including respiratory frequency (F), tidal volume (TV), and minute ventilation (MV) were recorded over a 10-min interval. These measurements were averaged by the system software (FinePointe v2.3.1.16; Buxco/DSI) and imported into Microsoft Excel for statistical analysis.

### Statistical analysis

For immunostaining and Western blotting, statistical analysis was performed by one-way analysis of variance (ANOVA) followed by Bonferroni’s multiple comparison test to determine significant changes between groups using Prism, version 9.4.0 (GraphPad Software Inc., San Diego, CA, United States). Data are reported as the mean ± SD and the number (n) of independent experiments. For ANOVA analyses, a *p-*value of less than 0.05 was considered significant.

## Results

### Further knockout of lipin1 in dystrophic diaphragm worsens diaphragm histopathology

**FIGURE 1:**
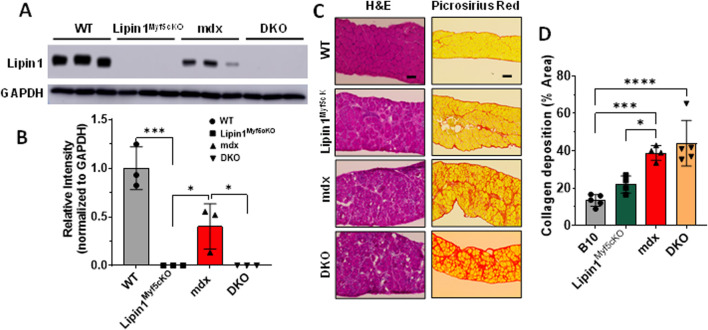
Histology analysis of the diaphragm muscles of WT, lipin1^*Myf5cKO*^, mdx, and dystrophin/lipin1-DKO mice. **(A)** Representative Western blot and **(B)** densitometric analysis of lipin1 in the diaphragm muscles of WT, lipin1^*Myf5cKO*^, mdx, and DKO mice (n = 3 mice/group). **(C)** representative H&E and Sirius red staining images, and **(D)** collagen deposition quantification analysis of diaphragm transverse sections (n = 5-6 mice/group). Scale bar = 200 μm. *p < 0.0001.

To assess the role of lipin1 in dystrophic diaphragm muscle, we employed *dystrophin:lipin1-DKO (DKO)* mouse model (Jama et al., 2023). The protein expression levels of lipin1 in the diaphragm of 6-month-old B10 wildtype *(WT)*, skeletal muscle-specific lipin1 knockout (*lipin1* ^*Myf5cKO*^), *mdx*, and *DKO* mice were measured by Western blotting. While the *lipin1* ^*Myf5cKO*^ and *DKO* mice did not show any lipin1 expression, the *mdx* diaphragm exhibited a 60% reduction of lipin1 expression compared to the *WT* diaphragm (*p = 0.007*, Figures 1A,B), which is consistent with our previous study (Jama et al., 2023). We compared the histopathological changes in the diaphragm of *WT*, *lipin1* ^*Myf5cKO*^, *mdx*, and *DKO* mice at 6 months of age. Hematoxylin and eosin (H&E) staining revealed mild inflammation infiltration and fibrosis in *lipin1* ^*Myf5cKO*^ mice, but some areas along the *mdx* and *DKO* mouse diaphragm cross-section were completely overridden by pale fibrotic tissue (Figure 1C).

As increased fibrosis results in elevated muscle stiffness and contributes to respiratory failure, we also evaluated fibrosis in *mdx* and *DKO* mice at 6 months old using Picrosirius red staining. In comparison to the *WT* diaphragm, the *lipin1* ^*Myf5cKO*^ diaphragm presented a 60% increase in collagen deposition. Collagen deposition was further exacerbated in the *mdx* and *DKO* diaphragm, which showed nearly a 2.9- and 3.2-fold increase (*p = 0.0005 and p < 0.0001*), respectively in comparison to the *WT* diaphragm (Figures 1C,D).

### Further knockout of lipin1 in the dystrophic diaphragm leads to enhanced cell death, inflammation, and fibrosis

**FIGURE 2:**
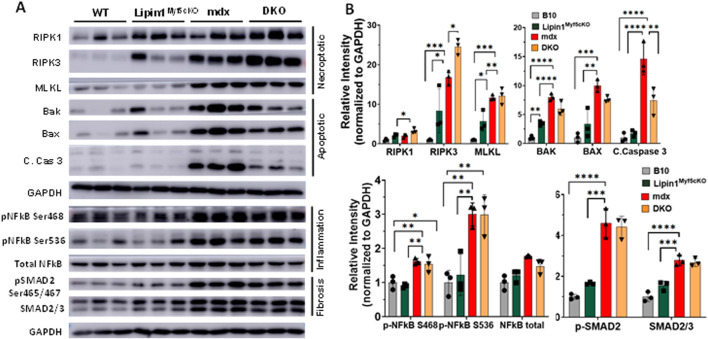
Protein expression levels of cell death, inflammation, and fibrosis markers in the diaphragm muscles of WT, lipin1^*Myf5cKO*^, mdx, and dystrophin/lipin1-DKO mice. **(A)** Western blot and **(B)** densitometric analysis of necroptotic, apoptotic, inflammation, and fibrosis markers in the diaphragm of WT, lipin1^*Myf5cKO*^, mdx, and DKO mice (n = 3 mice/group). *p < 0.0001.

Necroptosis, a form of regulated necrotic cell death, is mediated by receptor-interacting serine/threonine-protein kinase 1 (RIPK1), RIPK3, and mixed-lineage kinase-domain-like pseudokinase (MLKL) which contribute to muscle degeneration (Morgan et al., 2018). In DMD, RIPK3 is thought to be the major driver of limb muscle degeneration (Morgan et al., 2018). To identify the role of lipin1 overexpression in diaphragm muscle degeneration, we assessed these necroptosis markers in the diaphragm of 6-month-old *B10 WT*, *lipin1* ^*Myf5cKO*^, *mdx*, and *DKO* mice (Figures 2A,B). We found that RIPK1 and RIPK3 were significantly increased by 2- and 16-fold *(p = 0.025 and p = 0.001)* respectively, in *mdx* and further increased by 3- and 25-fold (*p = 0.0005 and p < 0.0001*) respectively in *DKO* compared to the *WT* diaphragm (Figures 2A,B). Although necroptotic markers were elevated in the *dystrophin/lipin1-DKO* diaphragm compared to the *mdx* diaphragm, we did not find significant differences in apoptotic cell death markers between these two groups. It is likely that necroptosis rather than apoptosis is the main cell death pathway contributing to the tissue damage observed by further knockdown of lipin1.

NF-kB is a master transcriptional factor regulating inflammation response. Phosphorylation of NF-kB at Ser468 and Ser536 has been shown to stimulate NF-κB transcriptional activity (Viatour et al., 2005; Campbell and Perkins, 2004; Oeckinghaus et al., 2011). We found that both Ser468 and Ser536 of NF-kB were highly increased in the *mdx (p = 0.005 and p = 0.004)* and *DKO* (*p = 0.011 and p = 0.004*) diaphragms compared to the *WT* diaphragm (Figures 2A,B). We did not find a further activation of NF-kB in the *DKO* diaphragm compared to the *mdx* diaphragm. Since increased SMAD signaling has been shown to drive the expression of extracellular matrix components such as collagens (Walton et al., 2017), we measured the protein expression levels of SMAD2/3 and found that protein expression levels were elevated by 2.7-fold in *mdx* and *DKO* diaphragms compared to *WT* diaphragm (*p < 0.0001*). Phosphorylation of SMAD2 at Ser465 and Ser467 promotes SMAD activation (Souchelnytskyi et al., 1997). Phospho-SMAD2 was also substantially elevated in *mdx* and *DKO* diaphragms compared to *WT* controls (*p < 0.0001 and p < 0.0001, respectively*). We did not observe any difference in protein expression levels of SMAD2/3 and pSMAD2 between the *mdx* and *DKO* diaphragms.

**FIGURE 3:**
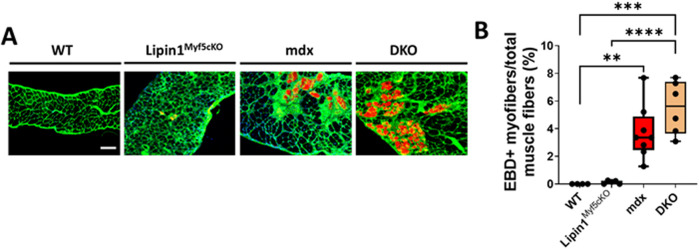
Muscle damage in the diaphragms of WT, lipin1^*Myf5cKO*^, mdx, and dystrophin/lipin1-DKO mice. **(A)** Representative images of EBD uptake (red) in diaphragm muscle sections of WT, lipin1^*Myf5cKO*^, mdx, and DKO mice. WGA (green) was used to visualize the borders of muscle fibers and DAPI (blue) stained nuclei. **(B)** Quantification analysis of EBD-positive muscle fiber expressed as the percentage of the total number of muscle fibers in each mouse (n = 4−8 mice/group). Scale bar = 200 μm. **p < 0.01; ***p < 0.001; ****p < 0.0001.

To determine whether knockout of lipin1 affects diaphragm muscle damage in mdx mice, we injected EBD into *WT*, *lipin1* ^*Myf5cKO*^, *mdx*, and *DKO* mice. Compared to *WT* controls, muscle damage was observed in both *mdx* and *DKO* diaphragms, but we did not observe any difference in muscle damage between *mdx* and *DKO* diaphragms (Figure 3). Wheat germ agglutinin (WGA) staining was used to identify the myofiber borders.

### Restoration of lipin1 in mdx:lipin1^Tg/0^ mice prevented pathology of the dystrophic diaphragm

**FIGURE 4:**
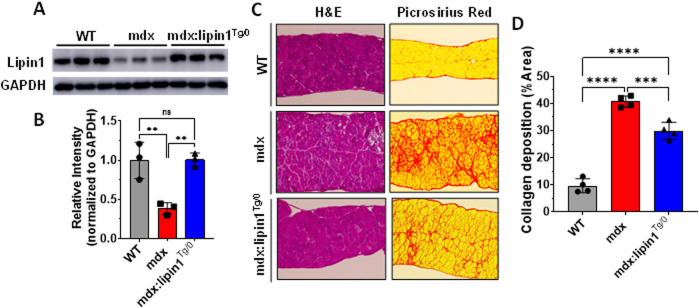
Histology analysis of the diaphragm muscles of WT, mdx, and mdx:lipin1^*Tg/0*^ mice. **(A)** Representative Western blot and **(B)** densitometric analysis of lipin1 in the diaphragm muscles of WT, mdx, and mdx:lipin1^*Tg/0*^ mice (n = 3 mice/group). **(C)** Representative H&E and Sirius red staining images, and **(D)** collagen deposition quantification analysis of diaphragm transverse sections (n = 4–5 mice/group). Scale bar = 200 μm. **p < 0.01; ***p < 0.001; ****p < 0.0001.

To evaluate whether increasing lipin1 expression levels in dystrophic diaphragm could suppress dystrophic pathology, we generated mice with lipin1 specifically overexpressed in skeletal muscles (Rosa26-lipin1^KI^) by crossing Rosa26-Stop-Lipin1 mice with mice carrying the Cre recombinase driven by the MCK gene promoter. We further crossed Rosa26-lipin1^KI^ with mdx mice, and generated mdx:lipin1 transgenic mice (*mdx:lipin1* ^*Tg/0*^) in which lipin1 was selectively increased in the dystrophic muscles of mdx mice, including in the diaphragm. We found that protein expression levels of lipin1 were substantially reduced in the diaphragm of mdx mice (*p = 0.007*, Figures 4A,B). Whereas, increasing lipin1 in hemizygous *mdx:lipin1* ^*Tg/0*^ mice restored lipin1 expression to its physiological levels similar to WT controls. Thus, hemizygous *mdx:lipin1* ^*Tg/0*^ mice have been used to examine the effect of lipin1 overexpression on DMD pathogenesis.

Muscle morphology analysis using H&E staining revealed decreased tissue damage in the lipin1-restored dystrophic diaphragm (Figure 4C). We also evaluated fibrosis in *mdx* and *mdx:lipin1* ^*Tg/0*^ mice at 6 months using Picrosirius red staining (Figures 4C,D). In comparison to the *mdx* diaphragm, the *mdx:lipin1* ^*Tg/0*^ diaphragm appeared to display substantially reduced fibrosis (*p = 0.0008*).

### Restoration of lipin1 in the dystrophic diaphragm suppressed cell death, inflammation, and fibrosis

**FIGURE 5:**
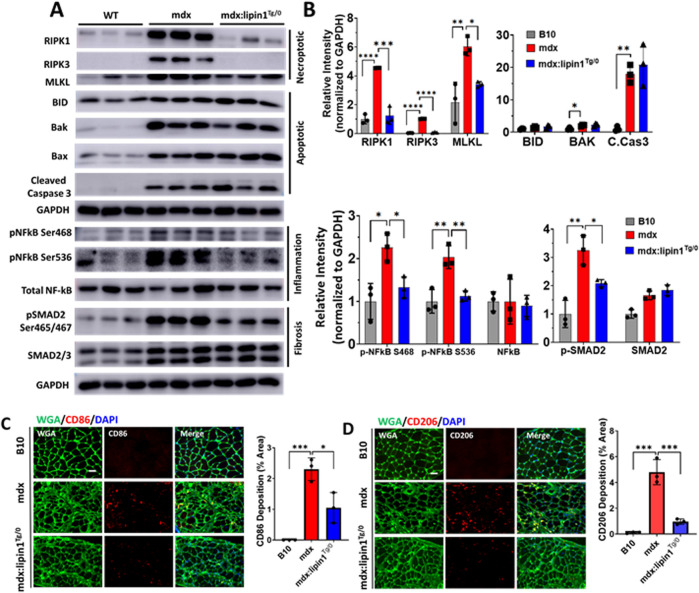
Protein expression levels of cell death, inflammation, and fibrosis markers in the diaphragm muscles of WT, mdx, and mdx:lipin1^*Tg/0*^ mice. **(A)** Western blot and **(B)** quantification analysis of necroptotic, apoptotic, inflammatory, and fibrosis markers in the diaphragm of WT, mdx, and mdx:lipin1^*Tg/0*^ mice. Representative immunostaining of **(C)** CD86+ (red) and **(D)** CD206+ (red) macrophages in diaphragms of indicated mice. WGA (green) was used to visualize the borders of muscle fibers and DAPI (blue) stained nuclei. Scale bar = 100 μm. n = 3 mice/group. *p < 0.05 **p < 0.01; ***p < 0.001; ****p < 0.0001.

To identify the role of lipin1 overexpression in diaphragm muscle degeneration, we assessed necroptotic markers in the diaphragm of 6-month-old hemizygous *mdx:lipin1* ^*Tg/0*^ and their *mdx* controls. As shown in Figures 5A,B, the expression levels of RIPK1, RIPK3, and MLKL were significantly elevated by 590%, 470%, and 310% (*p < 0.0001, p < 0.0001, and p = 0.005*) respectively in the *mdx* diaphragm compared to WT controls, but reduced to 26%, 41%, and 66% (*p = 0.0001, p < 0.0001, and p = 0.034*) respectively in the diaphragm of *mdx:lipin1* ^*Tg/0*^ compared to *mdx* mice, suggesting that increasing lipin1 expression levels inhibits necroptosis. The apoptotic markers BAK (*p = 0.02*), and cleaved-caspase-3 (*p = 0.004*) were elevated in the *mdx* diaphragm. However, restoration of lipin1 in dystrophic diaphragm did not reduce their expression levels compared to *mdx* diaphragm (Figure 5B) suggesting that restoration of lipin1 prevented the activation of necroptotic markers rather than apoptotic markers in dystrophic muscle.

Activation of NF-kB was measured to evaluate the effects of increasing lipin1 expression levels on inflammation in the dystrophic diaphragm. Western blot analysis showed that phosphorylation of NF-kB at serine 468 and 536 was elevated (*p = 0.0114 and p = 0.0031,* respectively) in the diaphragm muscle of mdx compared to *B10 WT* mice but was reduced to 59% and 55% (*p = 0.04 and p = 0.006*), respectively, in *mdx:lipin1* ^*Tg/0*^ compared to *mdx* mice (Figures 5A,B). Phosphorylation of SMAD2 was elevated by 420% in the *mdx* diaphragm and was reduced to 60% (*p = 0.0421*) when the expression of lipin1 was restored. Altogether this data suggests that restoration of lipin1 expression in the dystrophic diaphragm reduces cell death and inflammation, which ultimately leads to decreased collagen accumulation.

To further evaluate whether restoration of lipin1 could prevent inflammation, we also evaluated inflammatory macrophage distribution in *B10*, *mdx,* and *mdx:lipin1* ^*Tg/0*^ mice using immunohistochemistry. CD86^+^ macrophages are proinflammatory M1 macrophages that play an important role in dystrophic muscle pathology (Mojumdar et al., 2014). CD206^+^ macrophages are M2 macrophages that promote fibrosis development (Coulis et al., 2023). The abundance and distribution of CD86^+^ and CD206^+^ macrophages were detected by immunostaining in *B10*, *mdx*, and *mdx:lipin1* ^*Tg/0*^ muscle (Figures 5C,D). The surface area per cross-sectional area of CD86^+^ M1 macrophage was increased to 2.3% (*p = 0.0005*) in *mdx* compared to *WT* muscle, but was reduced to 1.0% (*p = 0.01*) in *mdx:lipin1* ^*Tg/0*^ muscle (Figure 5C). Moreover, CD206^+^ M2 macrophage was increased to 4.8% (*p = 0.0001*) in *mdx* compared to 0.1% in WT muscle, but was reduced to 0.96% (*p* = 0.0005) in *mdx:lipin1* ^*Tg/0*^ compared to *mdx* muscle (Figure 5D).

### Restoration of lipin1 reduced diaphragm muscle damage

**FIGURE 6:**
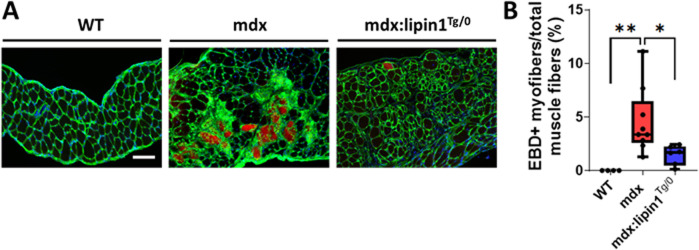
Muscle damage in the diaphragms of WT, mdx, and mdx:lipin1^*Tg/0*^ mice. **(A)** Representative images of EBD uptake (red) in diaphragm muscle sections of WT, mdx, and mdx:lipin1^*Tg/0*^ mice. WGA (green) was used to visualize the borders of muscle fibers and DAPI (blue) stained nuclei. Scale bar = 200 μm. **(B)** Quantification analysis of EBD-positive muscle fiber expressed as the percentage of the total number of muscle fibers in each mouse (n = 4−9 mice per group). *p < 0.05 **p < 0.01.

To determine whether restoration of lipin1 level alleviates muscle damage in *mdx* mice, we injected EBD into *WT*, *mdx*, and *mdx:lipin1* ^*Tg/0*^ mice. Compared to *mdx* littermates, *mdx/lipin1* ^*Tg/0*^ mice had much fewer EBD-positive muscle fibers (*p = 0.03*, Figure 6), suggesting that lipin1 upregulation increased sarcolemma stability and reduced muscle damage in *mdx* mice. These results suggest that compromised membrane integrity in dystrophic skeletal muscle can be ameliorated by lipin1 upregulation.

### Further knockout of lipin1 in the dystrophic diaphragm impaired respiratory function, and restoration of lipin1 improved respiratory function

**FIGURE 7:**
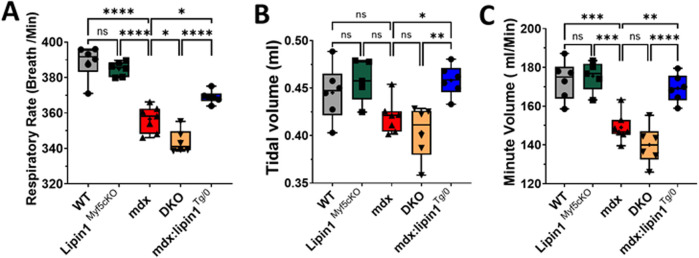
Respiratory function measurement of WT, lipin1^*Myf5cKO*^, mdx, DKO, and mdx:lipin1^*Tg/0*^ mice. **(A)** Respiratory rate, **(B)** tidal volume, and **(C)** minute volume in WT, lipin1^*Myf5cKO*^, mdx, DKO, and mdx:lipin1^*Tg/0*^ mice. All were measured by whole-body plethysmography. Respiratory measurements were taken over a 10-minute interval and averaged to obtain the data points for each mouse (n = 6–7 mice per group). *p < 0.05 **p < 0.01; ***p < 0.001; ****p < 0.0001.

Respiratory failure is a leading cause of death in DMD patients (Walton et al., 2017). Using whole-body plethysmography, we measured respiratory function in conscious, unrestrained 6-month-old *WT*, *lipin1* ^*Myf5cKO*^, *mdx*, *DKO,* and *mdx:lipin1* ^*Tg/0*^ mice. In the absence of just lipin1 in the lipin1^Myf5cKO^ mice, we did not observe any statistically significant respiratory impairment in comparison to the *B10 WT* mice (Figures 7A–C). We found that *mdx* mice had reduced respiratory rate (*p < 0.0001,* Figure 7A), and minute volume (*p = 0.0003,* Figure 7C) compared to *WT* mice, as in previous studies (Huang et al., 2011; Burns et al., 2015). Further knockout of lipin1 in dystrophic muscle led to a significantly reduced respiratory rate from 356 breaths/min to 344 breaths/min (*p = 0.01,* Figures 7A–C). The difference in tidal volume change between mdx mice and WT mice did not reach significance possibly due to sample variation, but the average tidal volume was reduced in mdx mice, which is consistent with a previous finding (Huang et al., 2011). There was not a significant difference in the tidal volumes and minute volumes between the *mdx* and *dystrophin/lipin1-DKO* mice most likely because mdx mice already present low levels of lipin1. In contrast, respiratory rate, and minute volume were all significantly improved in *mdx:lipin1* ^*Tg/0*^ compared to *mdx* mice (*p = 0.002,* Figures 7A–C), suggesting that increasing lipin1 expression levels improved respiratory function.

## Discussion

Respiratory failure is a leading cause of premature death in DMD patients. Although respiratory malfunction in DMD patients is slightly alleviated by noninvasive mechanical ventilation, identifying therapeutic targets and improving respiratory function would greatly impact DMD patients’ quality of life. In this study, we explored the roles of lipin1 in the dystrophic diaphragm and respiratory function using loss-of-function and gain-of-function approaches.

We identified that lipin1 protein expression levels were reduced by 60% in the *mdx* diaphragm. This is consistent with our previous study, which found that lipin1 decreased by 60% in the *mdx* gastrocnemius muscle compared to *WT* controls (Jama et al., 2023). Our recent studies revealed that lipin1 plays a complementary role in myofiber stability and muscle function in dystrophic muscles (Jama et al., 2023; Ramani et al., 2020; Jama et al., 2024). Lipin1 deficiency alone leads to compromised plasma membrane integrity, elevated necroptotic markers, and inflammation (Jama et al., 2023; Ramani et al., 2020). It is possible that lipin1 expression levels were already very low, so, further knockout of the remaining 40% of lipin1 in the dystrophic diaphragm did not lead to a significant worsening phenotype in this study. However, we did find that NF-kB, a master transcriptional factor of inflammation, was highly activated in the *mdx* and *DKO* diaphragms as indicated by enhanced phosphorylation of NF-kB at serine 468 and serine 536.

Dystrophin deficiency leads to an increase in muscle damage. The imbalance between muscle damage and repair is associated with apoptotic and necrotic cell death contributing to muscle fiber loss. Previous studies suggested that apoptosis characterizes the onset of the pathology of dystrophin-deficient muscle and precedes necrosis (Tidball et al., 1995). Apoptosis has been identified by nuclei with DMA fragmentation in the muscles of DMD patients and *mdx* mice (Tidball et al., 1995; Matsuda et al., 1995; Sandri and Carraro, 1999). Necroptosis, programmed necrosis, is distinguished from other modes of cell death in that it is highly proinflammatory given that cell membrane integrity is lost, triggering the activation of the immune system and inflammation. Therefore, necroptosis has been shown to be a major contributor to muscle degeneration in dystrophic muscles contributing to the loss of muscle fibers (Oeckinghaus et al., 2011). In our study, highly elevated Bax, Bak, and cleaved caspase 3 may indicate elevated apoptotic death in the *mdx* diaphragm. We observed that RIPK1, RIPK3, and MLKL are elevated in the diaphragm muscle of *mdx* mice, and RIPK1 and RIPK3 are further upregulated in the *DKO* diaphragm. Elevated necroptosis is not the only mechanism leading to muscle degeneration. Sarcolemmal disruptions, abnormal calcium (Ca^2+^) homeostasis (Culligan et al., 2002; Doran et al., 2006; Matsumura et al., 2009), increased calpain activities (Spencer and Mellgren, 2002), and NF-κB^38^ have been shown to contribute to muscle degeneration. The effects of loss of lipin1 in Ca^2+^ homeostasis and calpain activities remain to be investigated. Nevertheless, as a result of increased muscle degeneration, loss of lipin1 in the dystrophic diaphragm may lead to deterioration of respiratory function, indicated by a decrease in respiratory rate, which represents the most sensitive indicator of increasing respiratory difficulty.

Given the fact that loss of lipin1 plays an important role in dystrophic pathology, increasing lipin1 expression levels may have resulted in an improvement in muscle membrane integrity and respiratory function in *mdx* mice. Indeed, we found that the diaphragm of *mdx:lipin1* ^*Tg/0*^ mice had fewer EBD-positive muscle fibers compared to *mdx* controls, suggesting that compromised membrane integrity in the dystrophic diaphragm can be ameliorated by lipin1 restoration. This is consistent with our recent studies that lipin1 deficiency leads to increased sarcolemmal permeability, increased creatine kinase (CK) levels, and elevated necroptotic markers in animal models and cell culture systems (Jama et al., 2023; Jama et al., 2024). On the contrary, restoration of lipin1 in dystrophic muscle resulted in reduced sarcolemmal permeability, reduced serum CK levels, and downregulated necroptotic markers. Furthermore, restoration of lipin1 rescued the protein expression levels of α-sarcoglycan, dystroglycan, and nNOS, and promoted sarcolemmal stability (Jama et al., 2024).

Inflammation and cell death are effective hallmarks of membrane instability and dystrophin deficiency in DMD. NF-κB transcriptional activity is progressively elevated in DMD, and has been shown to contribute to disease onset and progression (Messina et al., 2011). We observed that the phosphorylation of NF-kB at serine 468 and serine 536 was suppressed in the diaphragm of *mdx:lipin1* ^*Tg/0*^ mice. In addition, CD86^+^ and CD206+ macrophages were highly elevated in mdx and DKO diaphragms compared to B10 controls suggesting that lipin1 restoration in dystrophic diaphragm significantly reduces myofiber inflammation. The protein expression levels of RIPK1, RIPK3, and MLKL were significantly suppressed in the diaphragm of *mdx:lipin1* ^*Tg/0*^ mice, suggesting that lipin1 restoration in dystrophic diaphragm reduced myofiber necrosis. Either inhibition of cell death or inflammation has been shown to improve dystrophic phenotype. Corticosteroids have been the first-line anti-inflammatory drugs approved by the FDA for the treatment of DMD. Inhibition of NF-κB in cardiomyocytes improved calcium handling and rescued cardiac function (Peterson et al., 2018). Genetic or pharmacologic inhibition of classical NF-κB DNA-binding subunit, p65, or its upstream activator, IκB kinase β (IKKβ), have been found to reduce inflammation and improve muscle regeneration in *mdx* mice (Acharyya et al., 2007). Genetic depletion of RIPK3 in dystrophic mdx mice reduced muscle degeneration and improved motor function (Morgan et al., 2018).

The ensuing loss of myofibers, largely mediated by a necrotic cell death process, is associated with the progressive replacement of the myofibers by fibrosis. Overall, our data suggest that the *mdx* diaphragm showed enhanced fibrosis indicated by Sirius red staining and activation of SMAD2. Activation of SMAD signaling via the canonical TGFβ pathway has been shown to promote collagen deposition and fibrosis (Coulis et al., 2023). Elevated diaphragm fibrosis results in muscle stiffness, prevents the diaphragm from achieving the excursion lengths required for respiration, and is the major contributor to the disease progression (Sahani et al., 1985). Increasing lipin1 expression in dystrophic muscle leads to substantially suppressed SMAD activation and reduced fibrosis development in *mdx:lipin1* ^*Tg/0*^ mice.

Patients with DMD experience progressive respiratory muscle weakness by 10–12 years of age (Spencer and Mellgren, 2002; Kumar and Boriek, 2003). As DMD progresses to later stages, patients often present a significant increase in respiratory rate, which is known as rapid shallow breathing (Lo Mauro and Aliverti, 2016). It has been suggested that rapid shallow breathing is adopted as a strategy to reduce the perception of labored breathing (Lo Mauro and Aliverti, 2016). We found that the respiratory rates, tidal volume, and minute volume were reduced in *mdx* and *mdx:lipin1-DKO* mice at 6 months of age measured by plethysmography. This is consistent with previous studies that *mdx* mice at 6 months old had significantly impaired respiratory function, indicated by reduced respiratory frequency, tidal volume, and minute volume (Huang et al., 2011). The impaired respiratory function is likely due to increased diaphragm sarcolemmal damage, inflammation, elevated cell death, and increased fibrosis which confer mechanical defects in the *mdx* diaphragm. We also found that restoration of lipin1 in the dystrophic diaphragm significantly improved the respiratory rate, tidal volume, and minute volume, suggesting that increasing lipin1 levels successfully improved respiratory function in *mdx* mice.

It should be noted that we observed only 4% of EBD^+^ myofibers in the skeletal muscle of *mdx* mice. The ratio was calculated by EBD^+^ myofiber areas *versus* the total muscle fiber area. The total muscle fiber areas were calculated by measuring the areas of the diaphragm ruling out the tissue gaps between borders of myofibers (Brown et al., 2023; Dubuisson et al., 2022). However, the amount of muscle damage does not seem to match the degree of impaired respiratory function and elevated cell death, inflammation, and fibrosis markers. These results suggest that the EBD staining may not represent muscle damage, but muscle sarcolemmal leakage. Although dystrophin-deficient muscle cells are vulnerable to membrane damage, not all muscle damage leads to sarcolemmal leakage when subjected to increased mechanical stress. Therefore, EBD staining needs to be combined with other techniques, such as plasma creatine kinase levels (Jama et al., 2023; Kobayashi et al., 2012), and the identification of intracellular fibronectin in muscle cells (Friden and Lieber, 1998) to provide a more comprehensive and integrated view of muscle damage. Indeed, in our recent study (Jama et al., 2024), we observed that *mdx* mice had elevated CK levels and increasing lipin1 expression levels in *mdx:lipin1* ^*Tg/0*^ mice reduced CK levels suggesting a critical role for lipin1 in maintaining myofiber stability and integrity. Another reason that leads to low EBD^+^ myofibers in 6-month-old *mdx* mice may be due to age. Previous studies found that EBD is significantly more abundant in the diaphragm of 4-week-old *mdx* than in the diaphragm of 24-week-old *mdx* mice (Pelosi et al., 2015). It is possible that younger *mdx* mice may exhibit more damaged muscle fibers. The variations of sarcolemmal leakage in *mdx* mice affected by age will need to be investigated in the future.

In conclusion, we found that lipin1 expression level is markedly reduced in the *mdx* diaphragm. Loss of lipin1 is associated with increased severity of pathology in the dystrophic diaphragm and contributes to respiratory dysfunction. In contrast, lipin1 overexpression significantly improves the primary defect and downstream pathology in the dystrophic diaphragm, resulting in improved membrane integrity, prevented inflammation, decreased muscle fiber degeneration, reduced fibrosis, and most importantly, improved respiratory function. These findings suggest that lipin1 is an important therapeutic target in the management and treatment of respiratory insufficiency and failure in DMD. In future studies, more functional assays will be introduced to highlight the protective effects of lipin1 on respiratory muscles. We will conduct *ex vivo* contractile assessments to measure the isometric force production of a thin diaphragm muscle strip. We will conduct exercise testing (e.g., running wheel; treadmill) on the *mdx:lipin1* ^*Tg/0*^, *mdx: mdx:lipin1-DKO,* and *mdx* mice. This type of stress will provide interesting discrimination from a whole-body functional perspective if lipin1 is overexpressed *versus* reduced in the *mdx* diaphragm. We will also assess whether lipin1 overexpression can ameliorate functional impairments and cellular markers of damage in a more severe mouse model (e.g., *D2.mdx* or *mdx-utrn dKO* mice). To explore the potential of lipin1 overexpression for treating respiratory failure, we will use an AAV-based strategy as a therapeutic tool to efficiently deliver lipin1 to the diaphragm. We will investigate the effectiveness, possible adverse effects, and long-term safety of different expression levels of lipin1 as a potential therapy for respiratory dysfunction in DMD. Another future direction is to investigate if older aged mice also present rapid shallow breathing with the progression of the disease; and if lipin1 restoration could help to mitigate this response. Moreover, continued investigation is required to better understand the mechanisms behind these findings, and to determine the role of lipin1 in maintaining muscle membrane stability.

## Data Availability

The datasets used and/or analyzed during the current study are available from the corresponding author upon reasonable request and directed to Hongmei Ren, hongmei.ren@wright.edu.

## Abbreviations

DMD, Duchenne muscular dystrophy; EBD, Evans blue dye; PA, phosphatidic acid; PAP, phosphatidic acid phosphatase; DAG, diacylglycerol; WT, wild-type; RIPK, receptor-interacting serine/threonine-protein kinase; MLKL, mixed-lineage kinase domain-like protein; OCT, optimal cutting temperature; WT, wildtype; MCK, muscle creatine kinase; CK, creative kinase.
