# Supplementary figures and images for: Lipin1 as a therapeutic target for respiratory insufficiency of duchenne muscular dystrophy
Source: Front Physiol. 2024 Nov 12;15:1477976. doi: 10.3389/fphys.2024.1477976 (PMC11588688; doi:10.3389/fphys.2024.1477976)

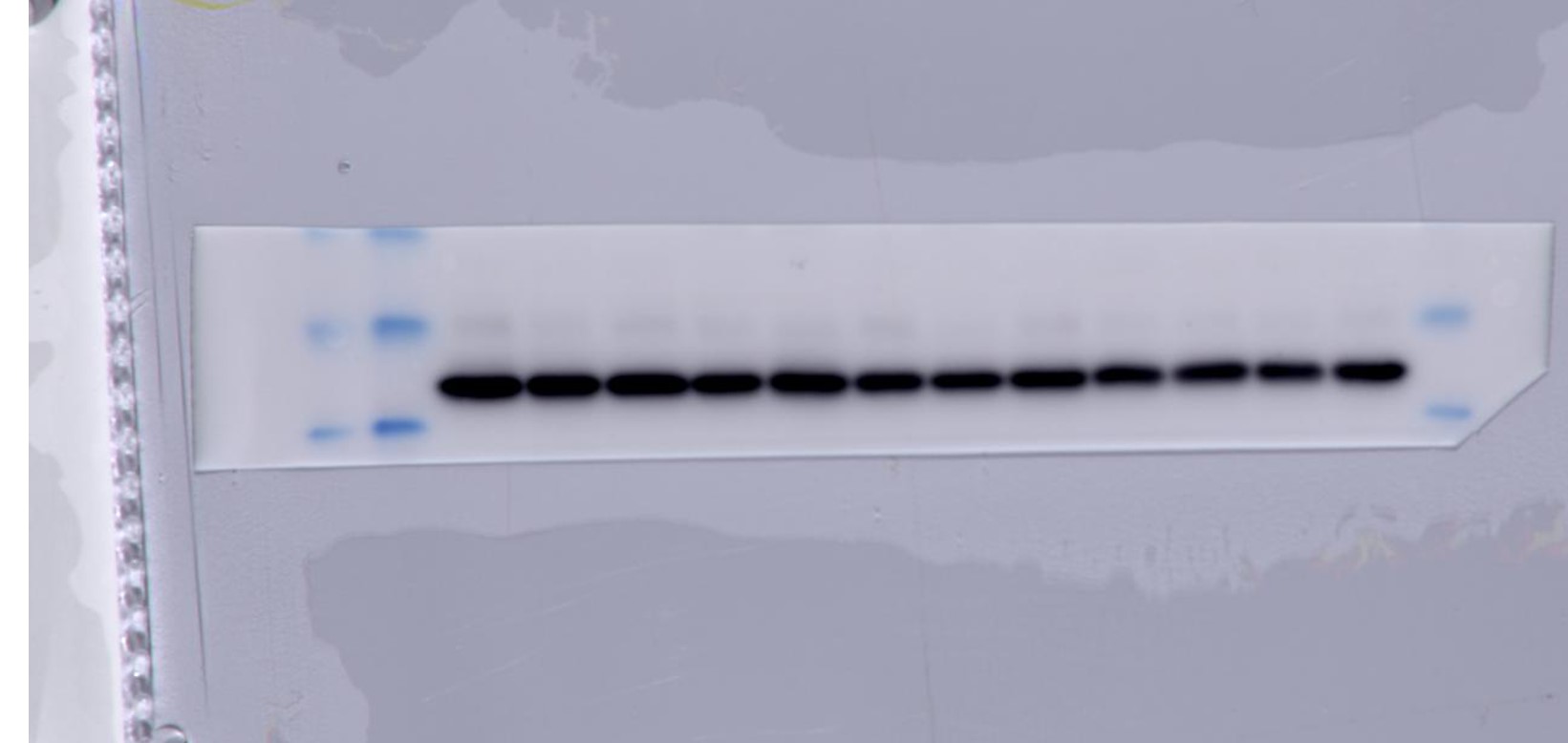

Supplement: Supplementary file 1 [file DataSheet1.zip › Western Raw Data/Figure 1/gapdh figure1.jpg]

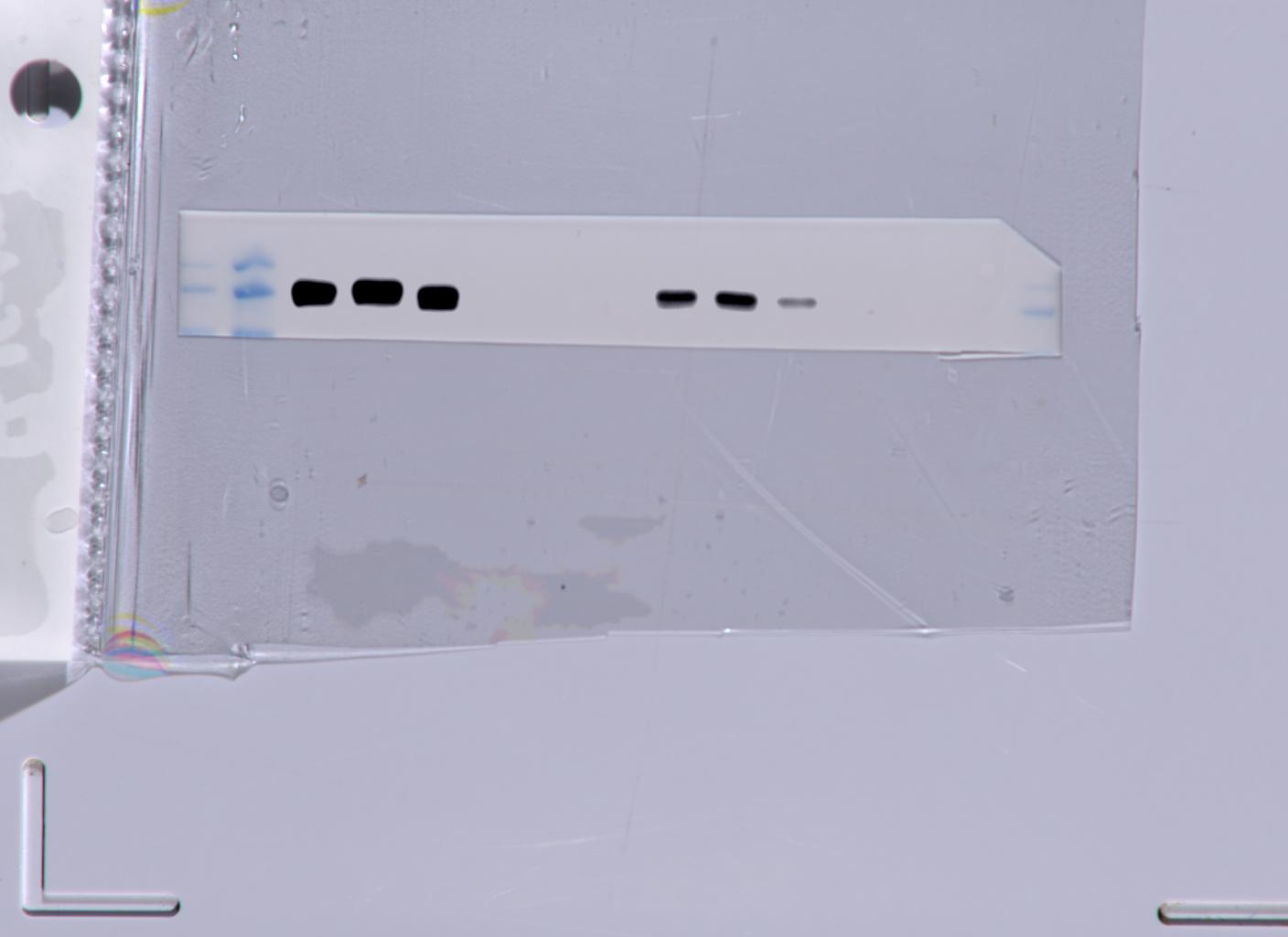

Supplement: Supplementary file 1 [file DataSheet1.zip › Western Raw Data/Figure 1/lipin1 Figure 1.jpg]

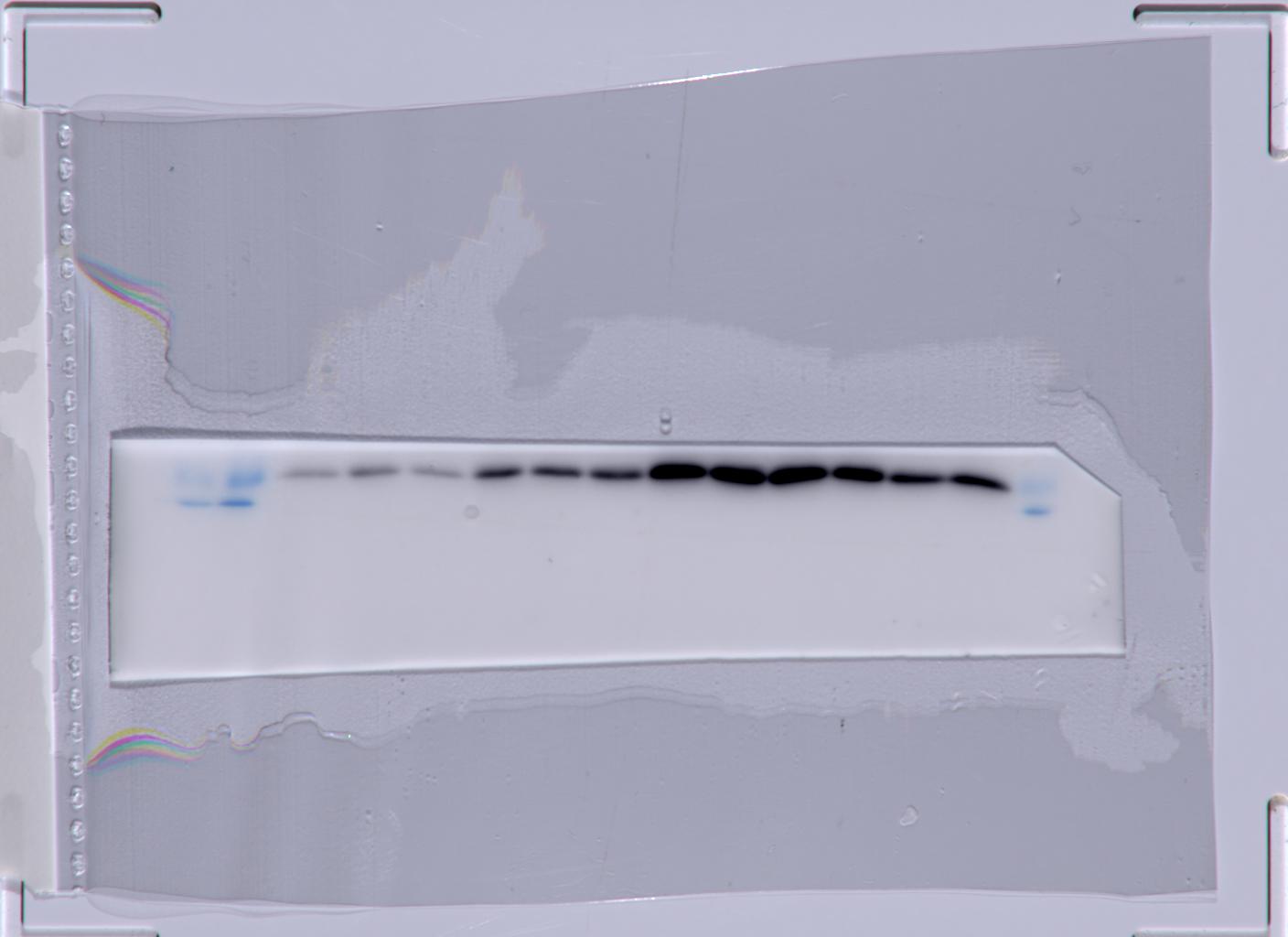

Supplement: Supplementary file 1 [file DataSheet1.zip › Western Raw Data/Figure 2/DKO Bak Figure 2.jpg]

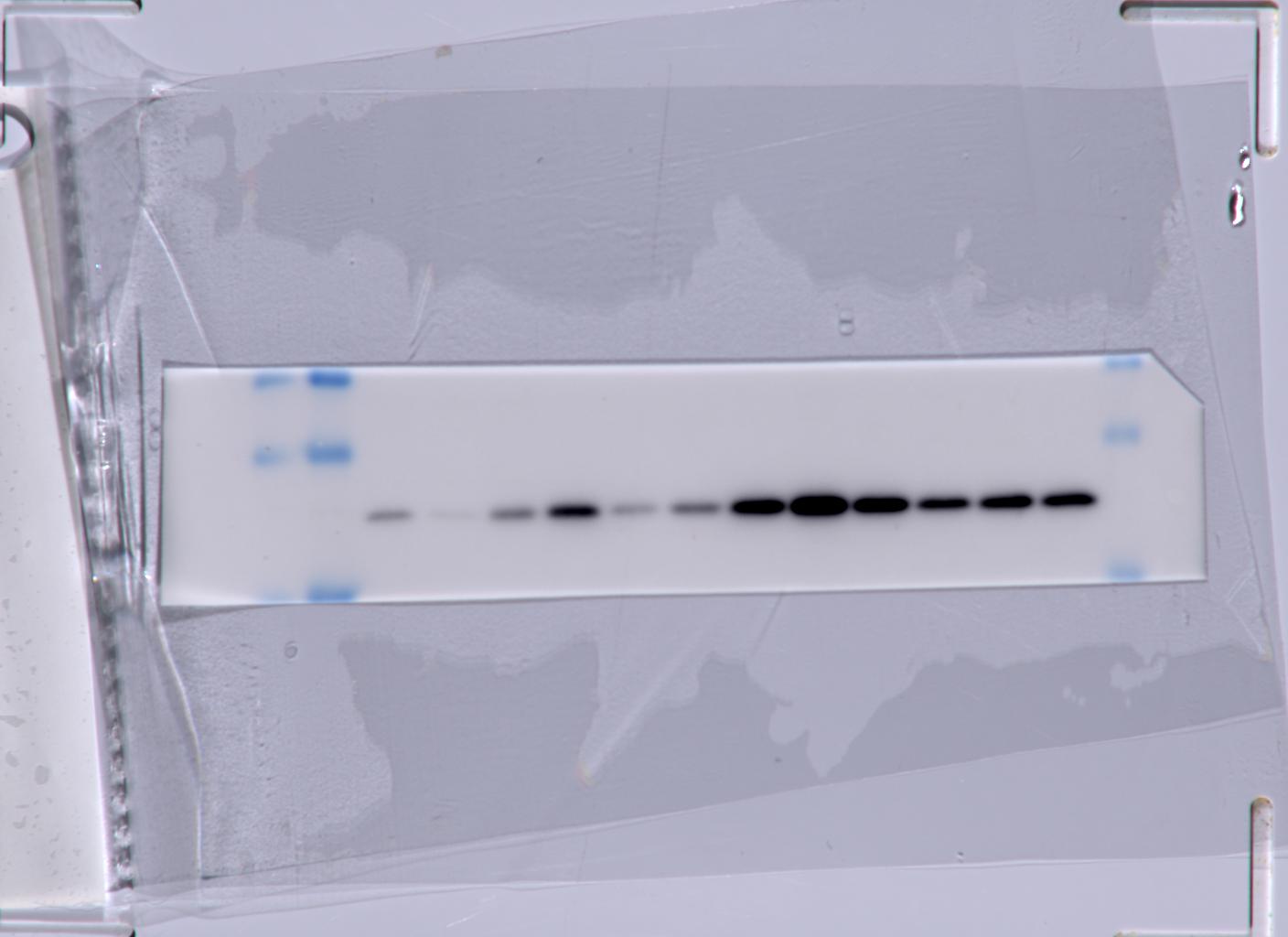

Supplement: Supplementary file 1 [file DataSheet1.zip › Western Raw Data/Figure 2/DKO BAX Figure 2.jpg]

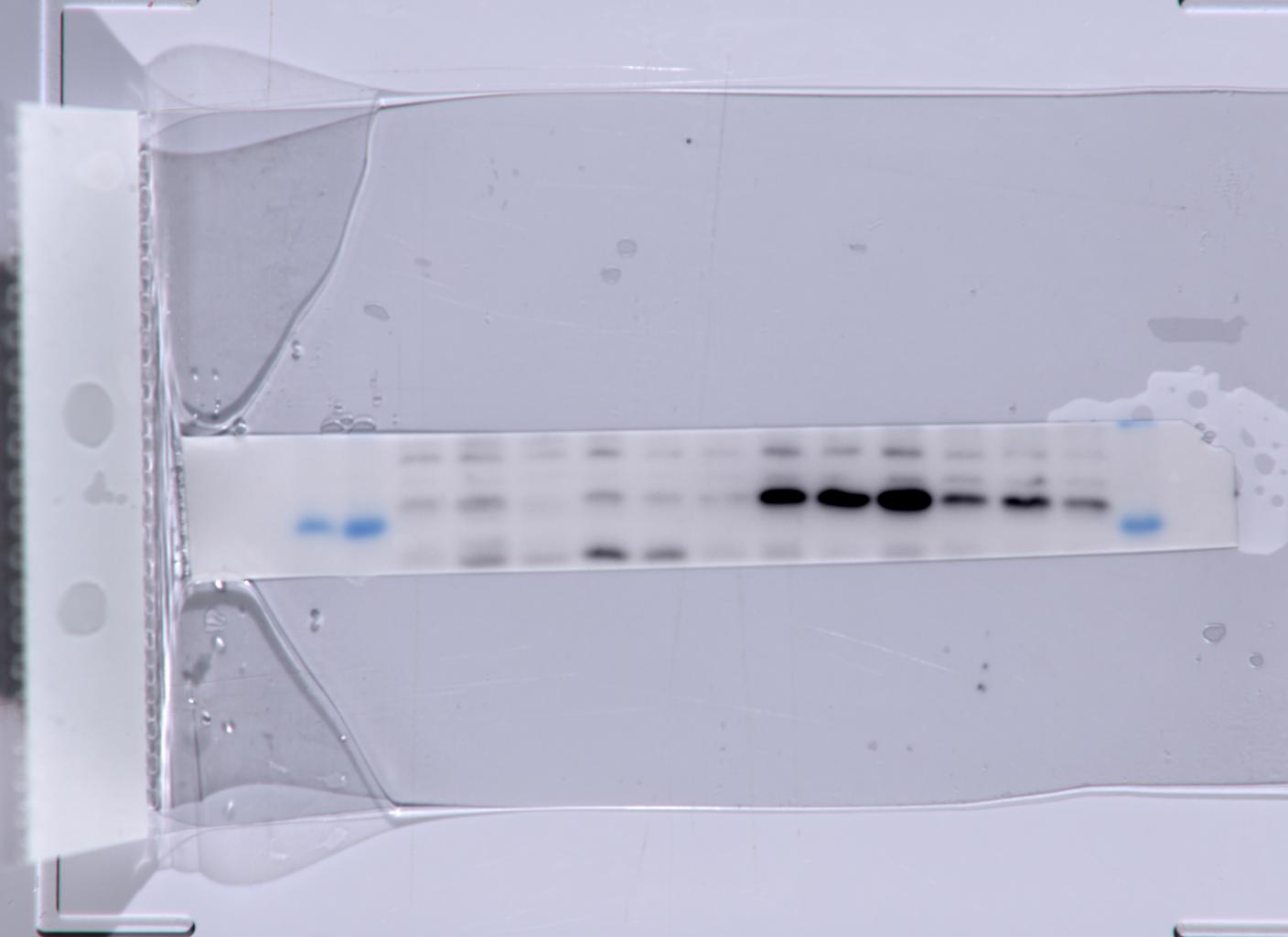

Supplement: Supplementary file 1 [file DataSheet1.zip › Western Raw Data/Figure 2/DKO CCas3 Figure 2.jpeg]

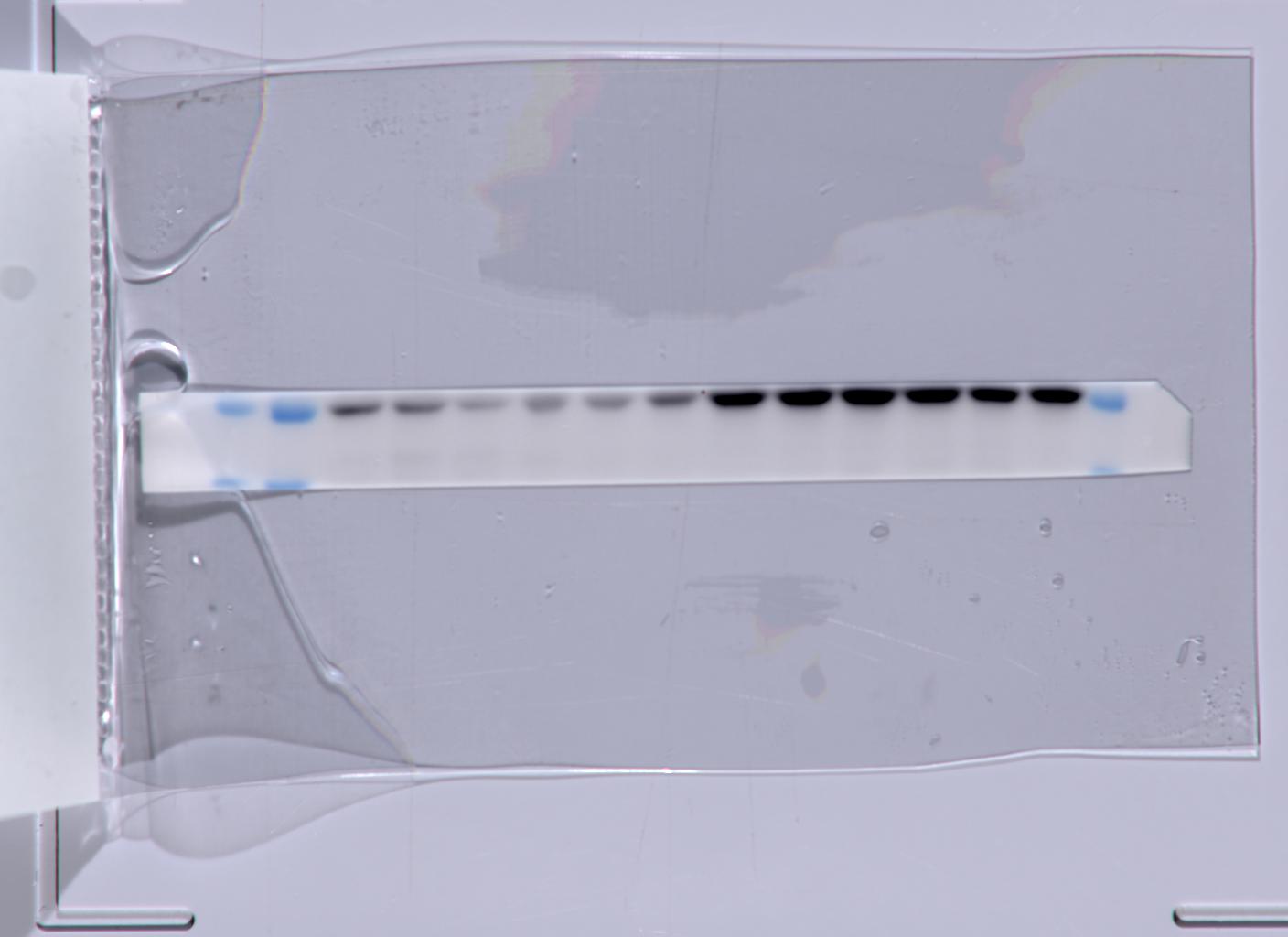

Supplement: Supplementary file 1 [file DataSheet1.zip › Western Raw Data/Figure 2/DKO MLKL Figure 2.jpg]

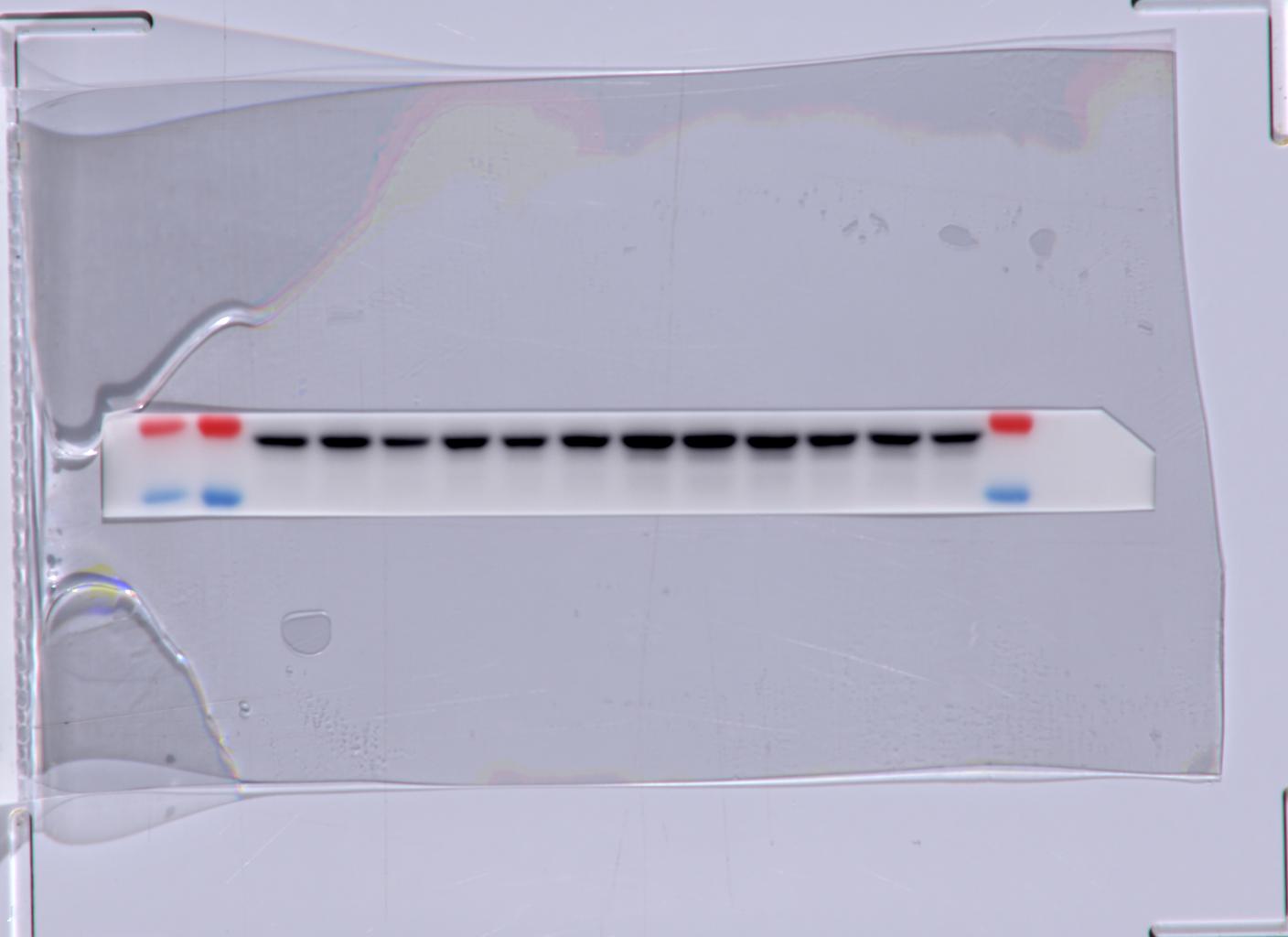

Supplement: Supplementary file 1 [file DataSheet1.zip › Western Raw Data/Figure 2/DKO NFkBtot Figure 2.jpeg]

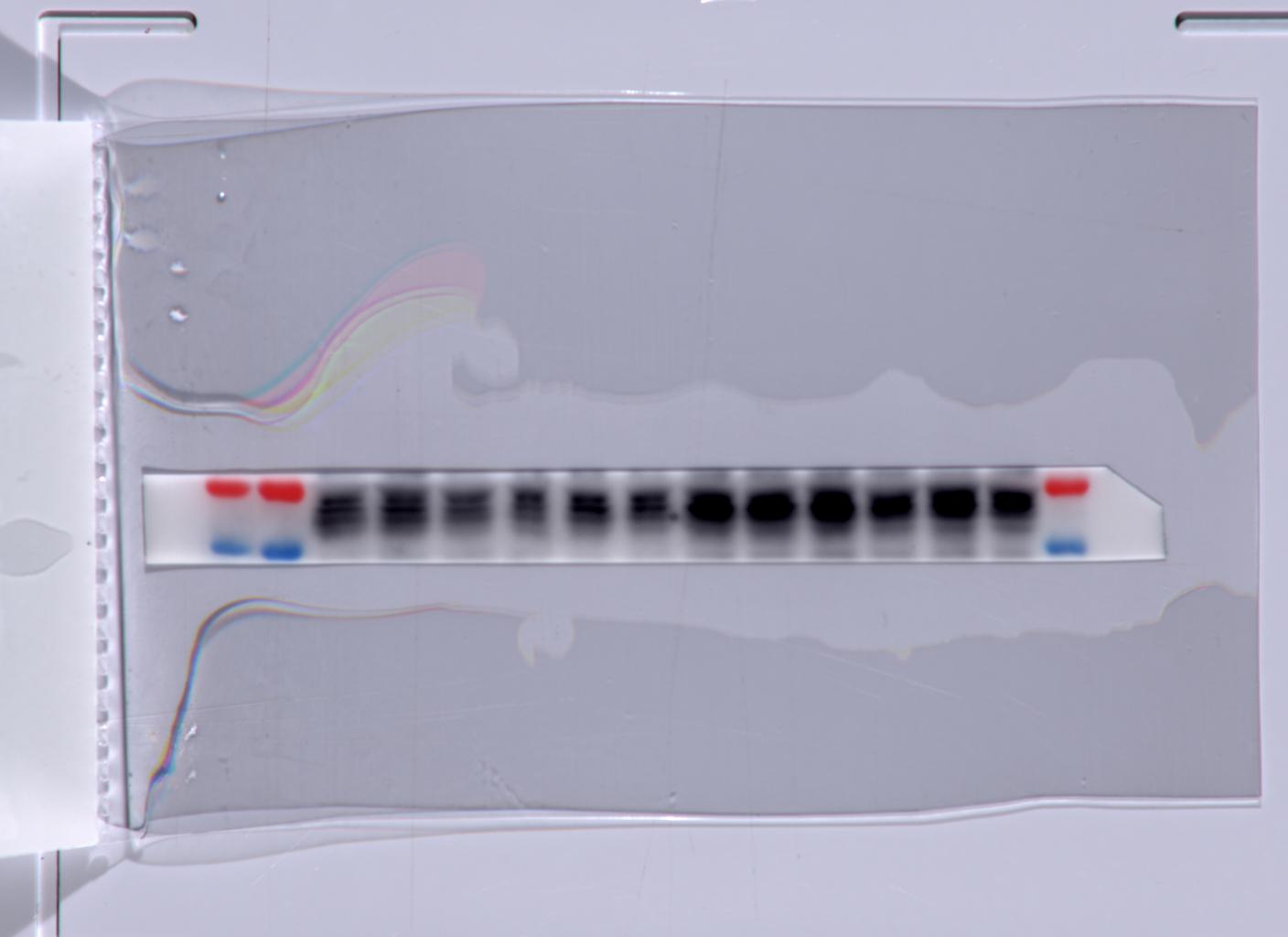

Supplement: Supplementary file 1 [file DataSheet1.zip › Western Raw Data/Figure 2/DKO pNFkBS468 Figure 2.jpg]

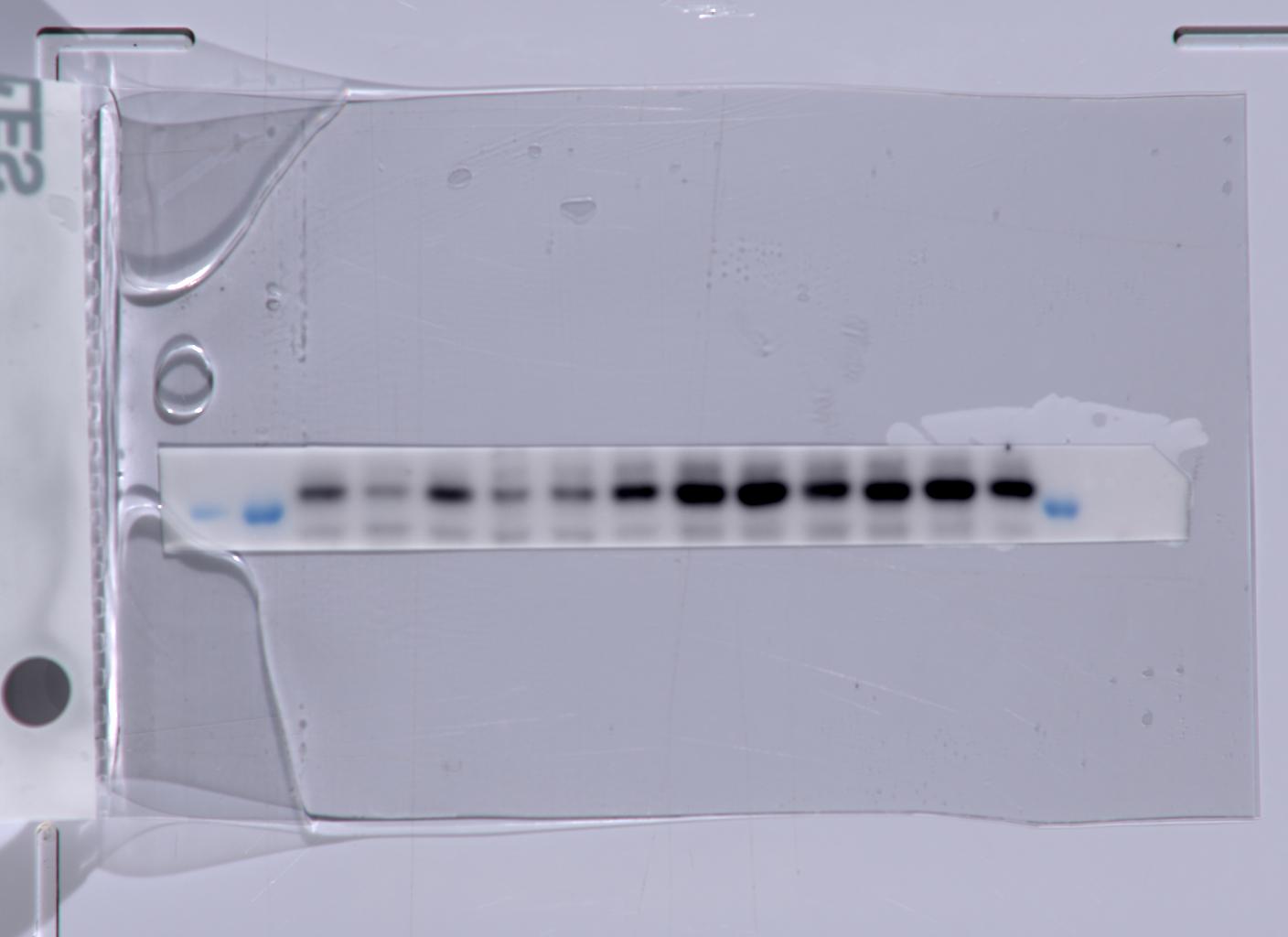

Supplement: Supplementary file 1 [file DataSheet1.zip › Western Raw Data/Figure 2/DKO pNFkBS536 Figure 2.jpeg]

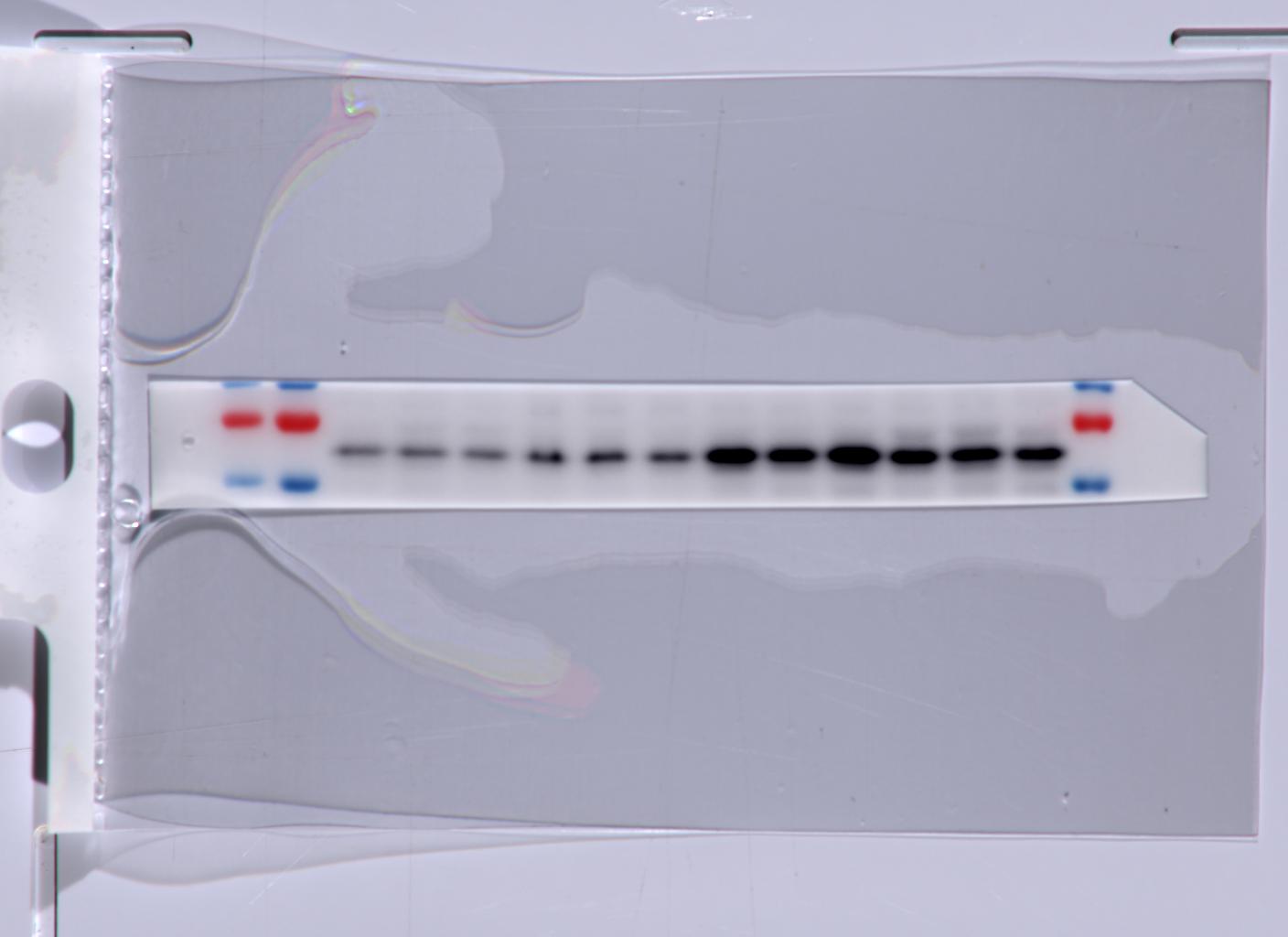

Supplement: Supplementary file 1 [file DataSheet1.zip › Western Raw Data/Figure 2/DKO pSMAD2 Figure 2.jpeg]

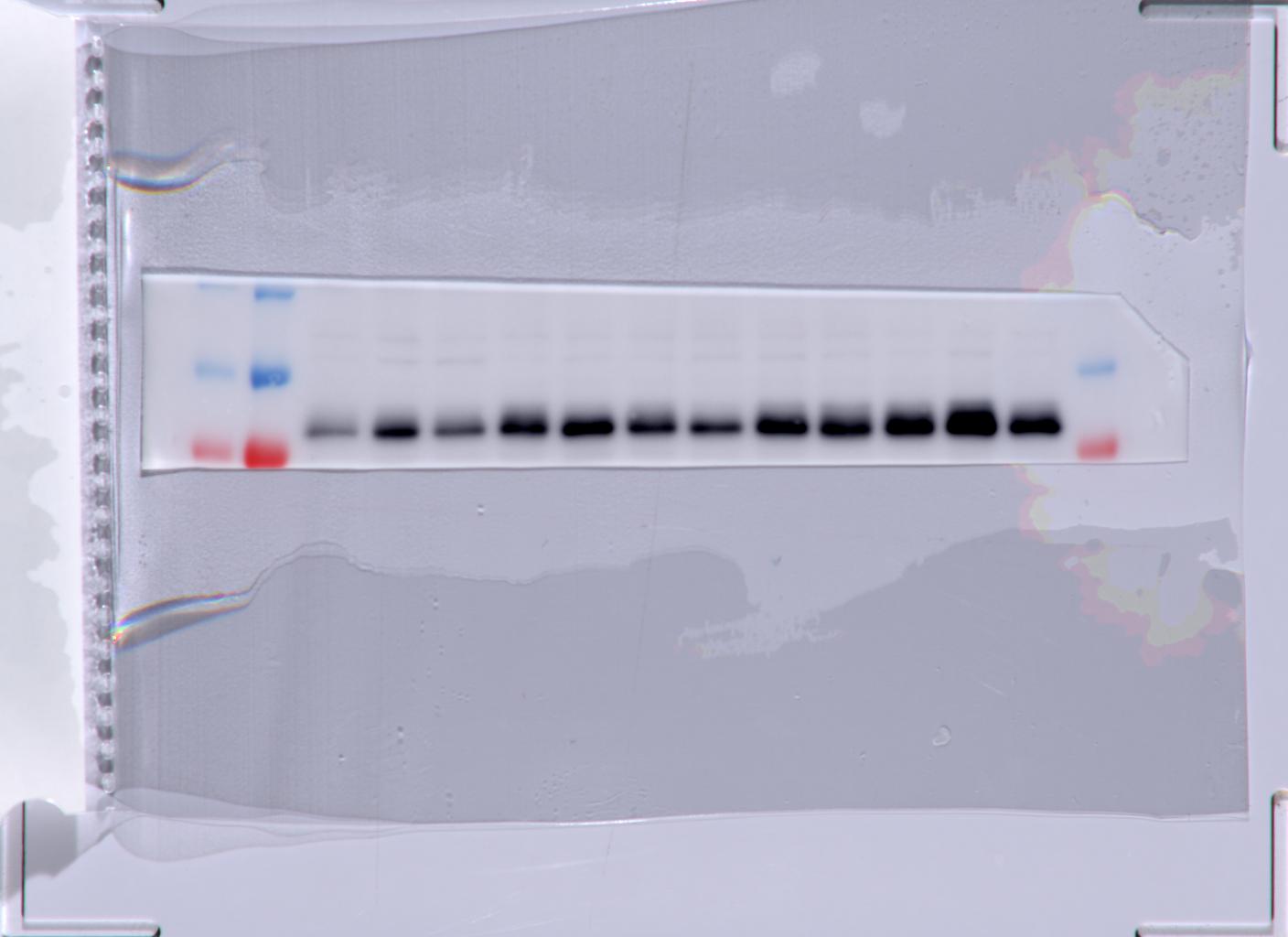

Supplement: Supplementary file 1 [file DataSheet1.zip › Western Raw Data/Figure 2/DKO RIPK1 Figure 2.jpg]

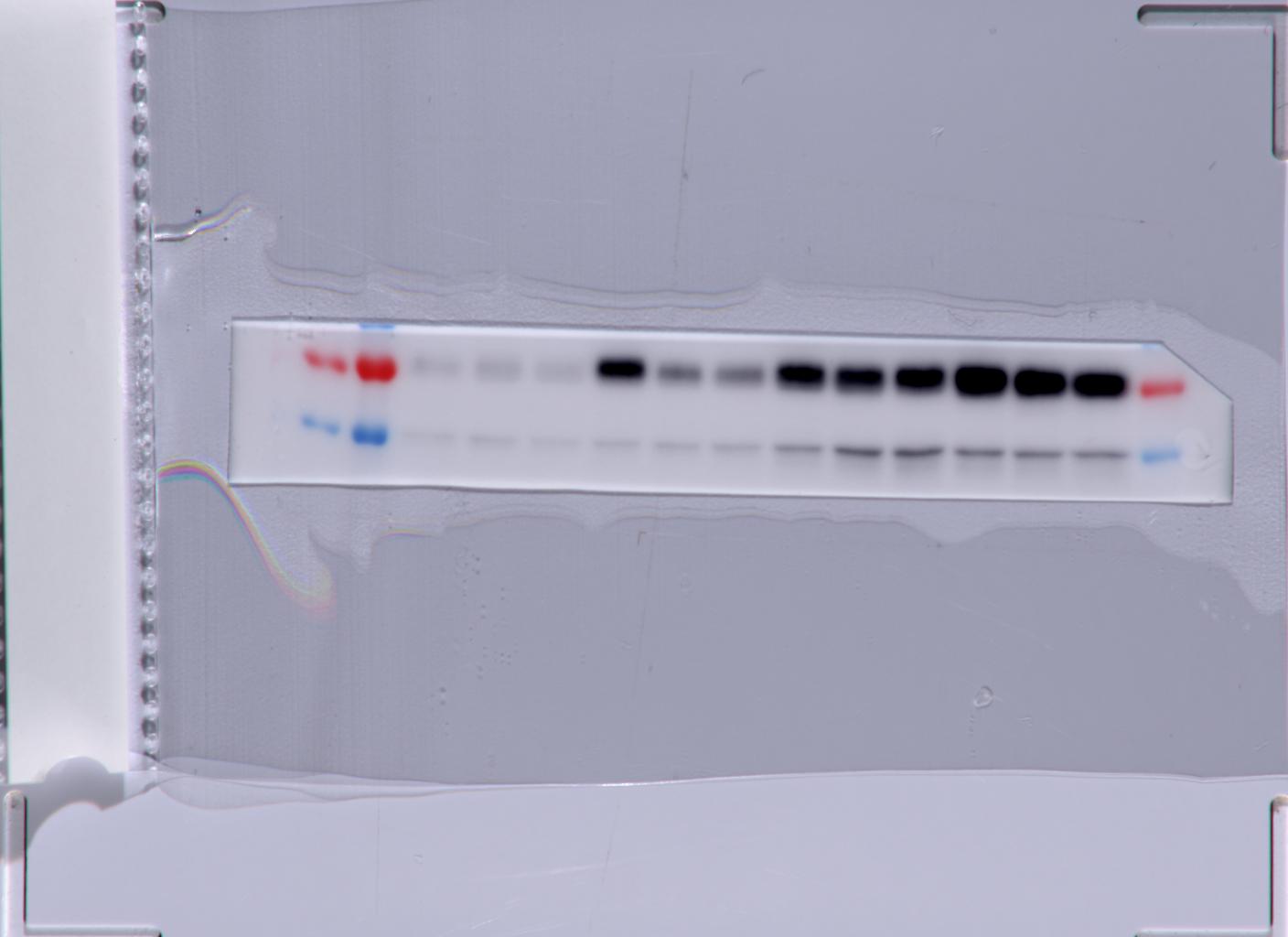

Supplement: Supplementary file 1 [file DataSheet1.zip › Western Raw Data/Figure 2/DKO RIPK3 Figure 2.jpg]

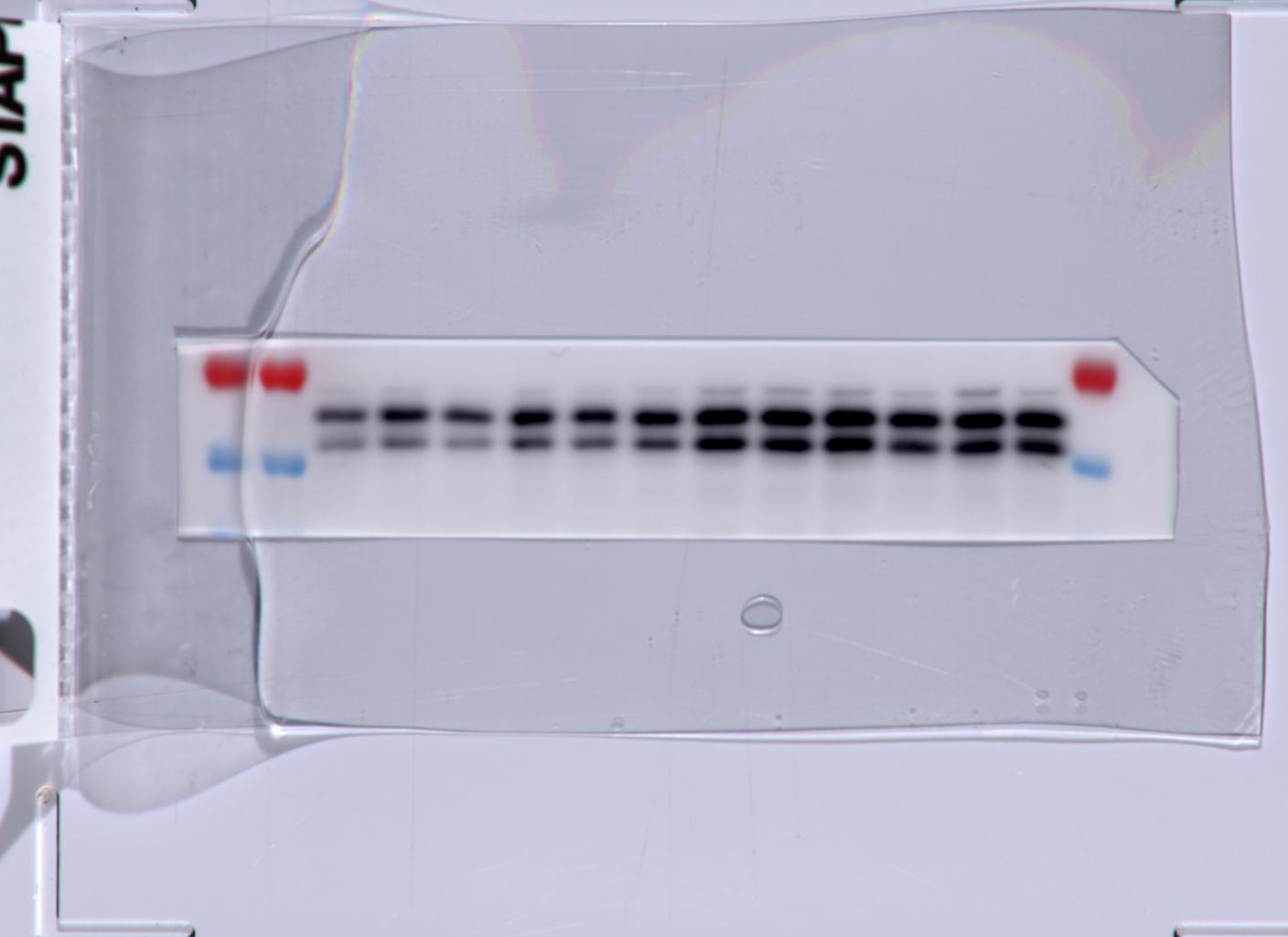

Supplement: Supplementary file 1 [file DataSheet1.zip › Western Raw Data/Figure 2/DKO SMAD2and3 Figure 2.jpeg]

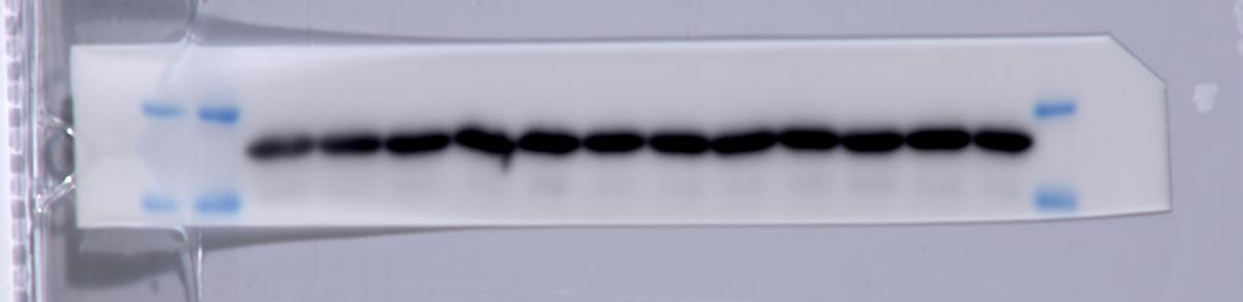

Supplement: Supplementary file 1 [file DataSheet1.zip › Western Raw Data/Figure 2/GAPDH Figure 2.png]

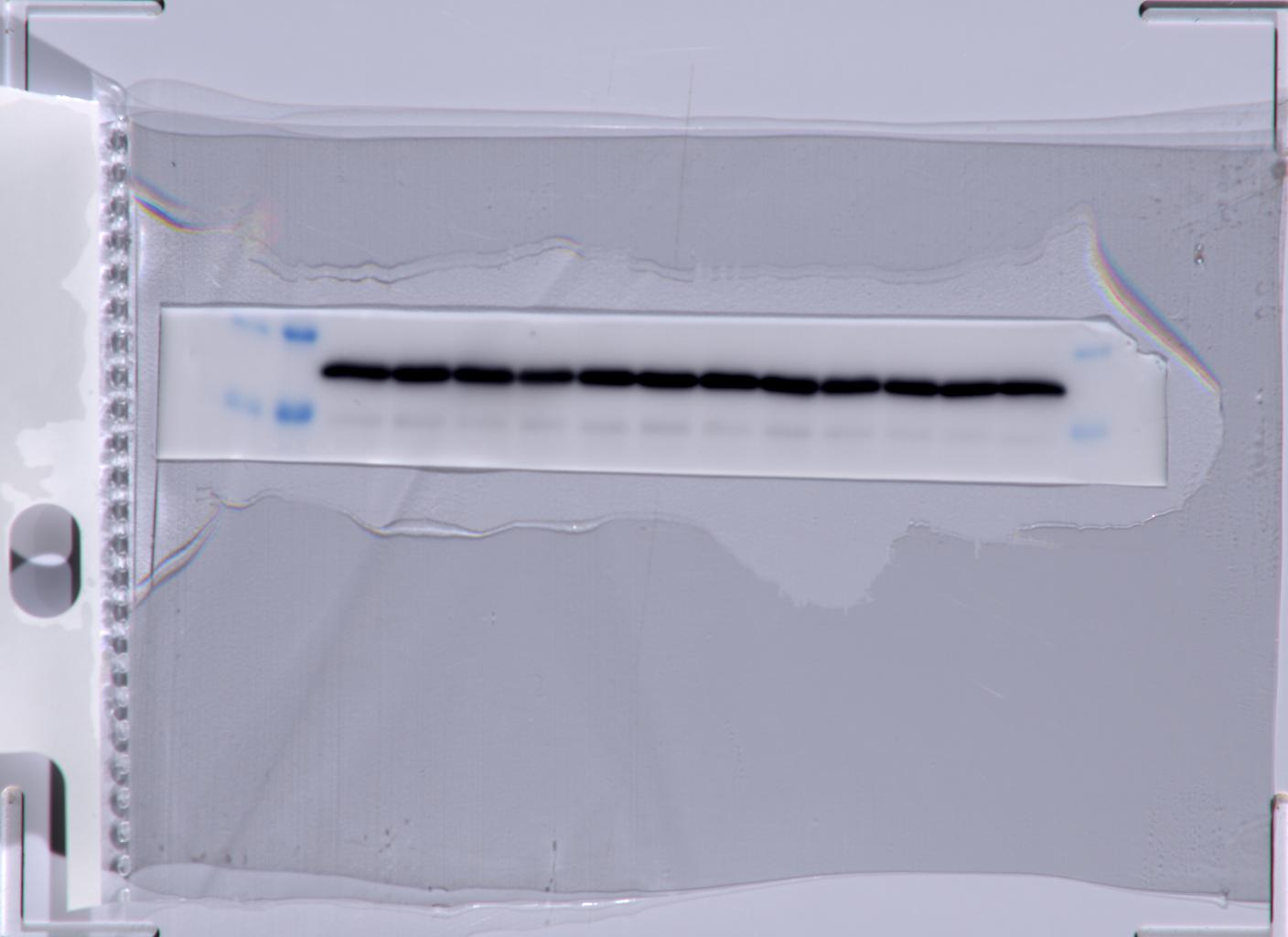

Supplement: Supplementary file 1 [file DataSheet1.zip › Western Raw Data/Figure 2/GAPDH2 Figure 2.jpg]

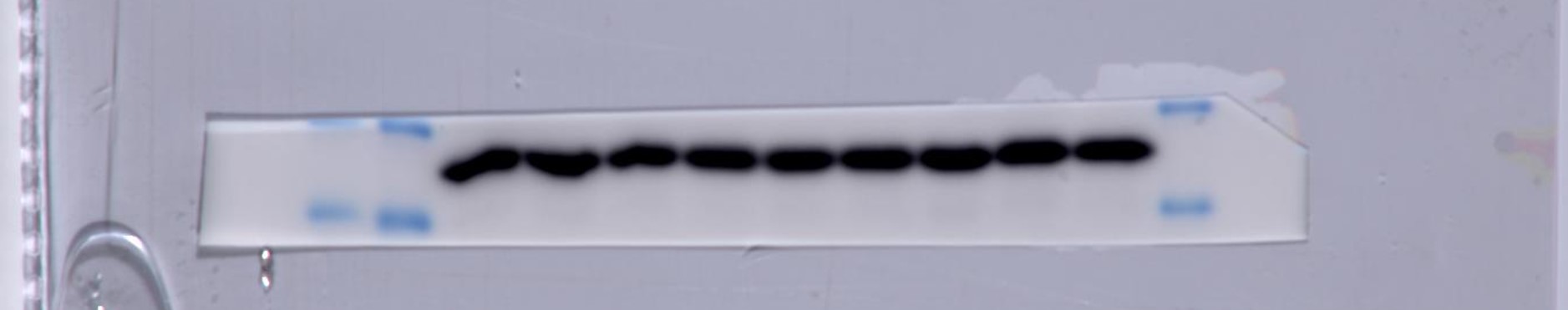

Supplement: Supplementary file 1 [file DataSheet1.zip › Western Raw Data/Figure 4/Cre GAPDH figure 4.jpg]

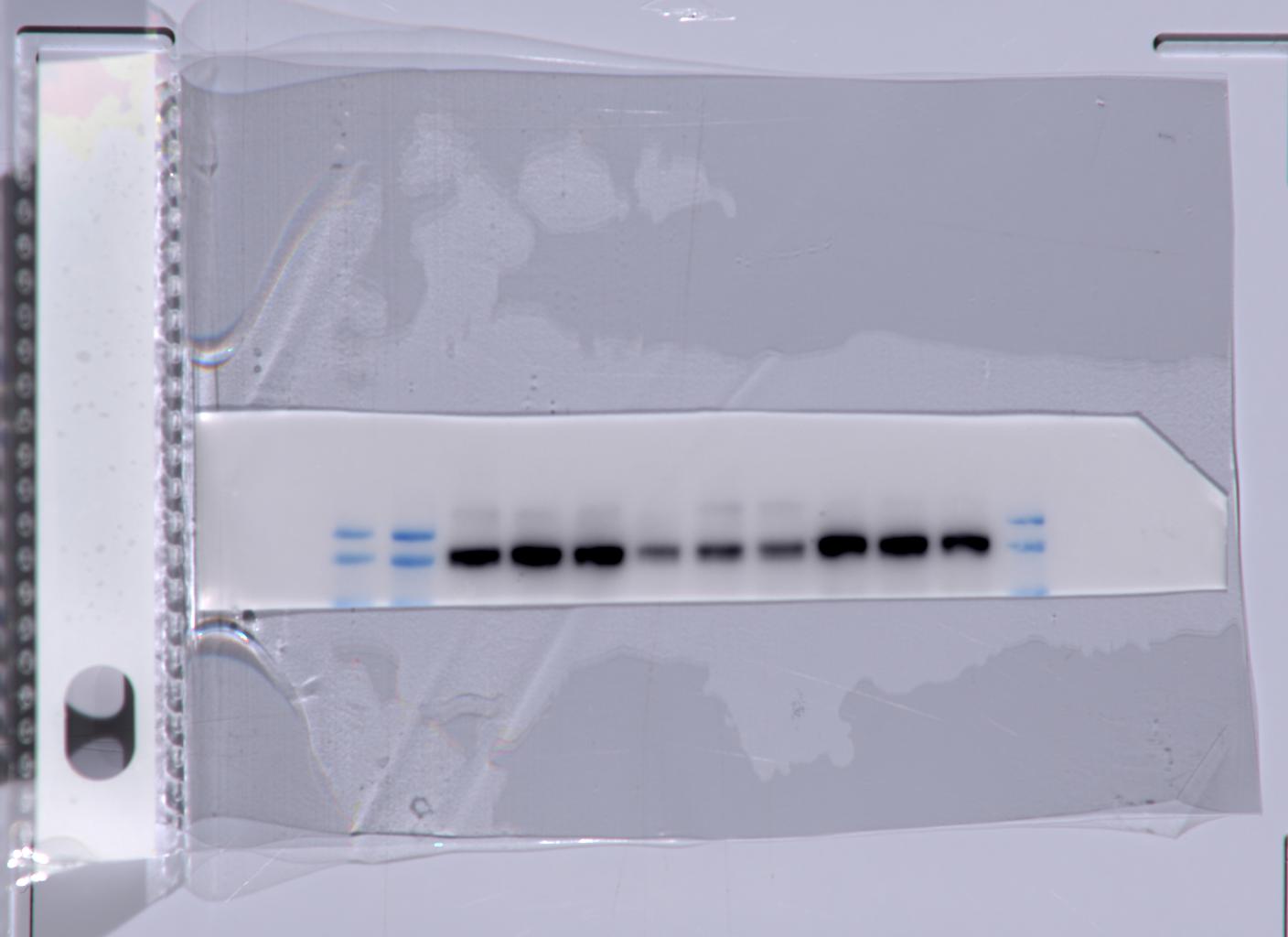

Supplement: Supplementary file 1 [file DataSheet1.zip › Western Raw Data/Figure 4/cre Lipin1 Figure 4.jpeg]

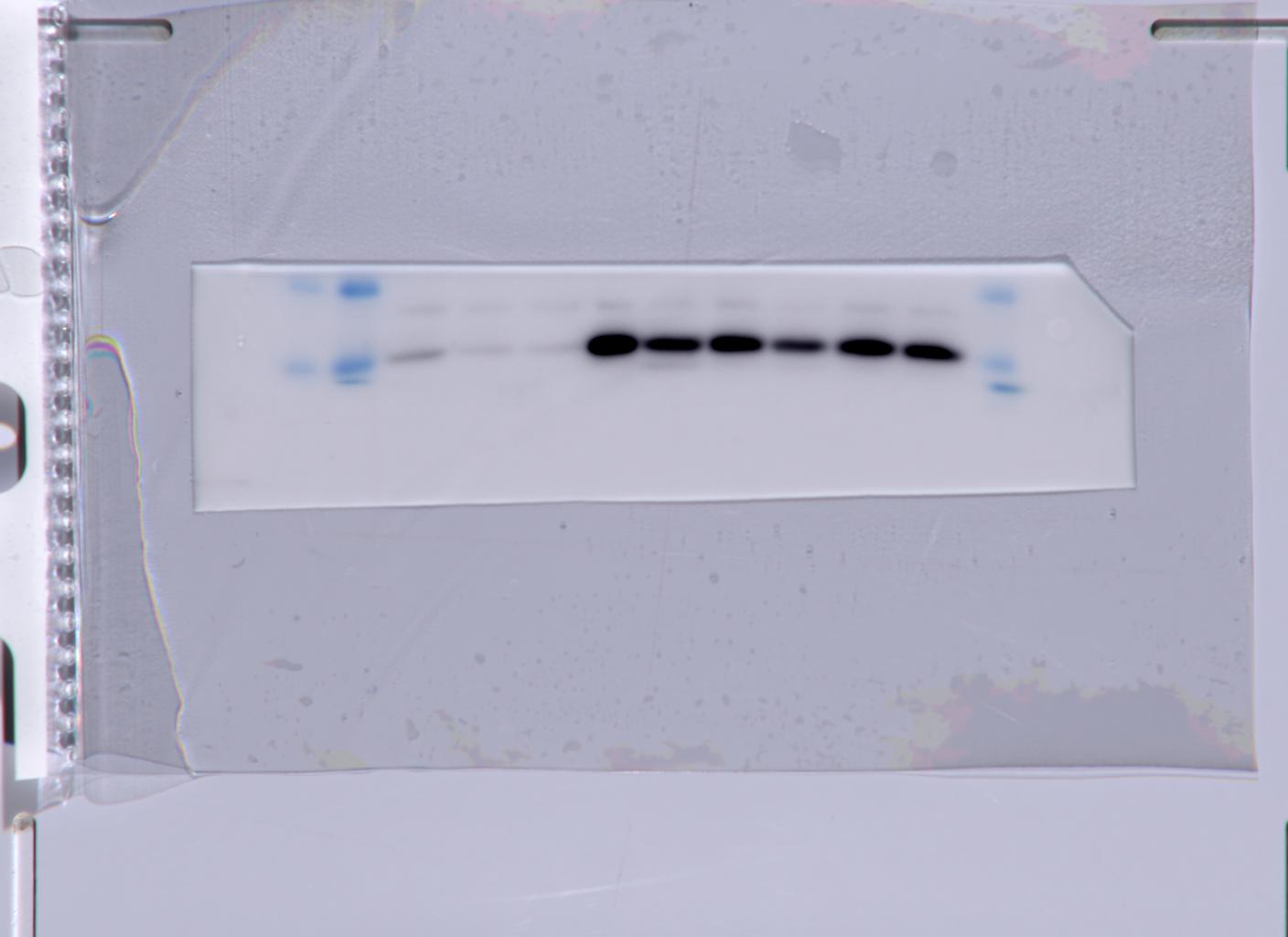

Supplement: Supplementary file 1 [file DataSheet1.zip › Western Raw Data/Figure 5/cre Bak Figure 5.jpeg]

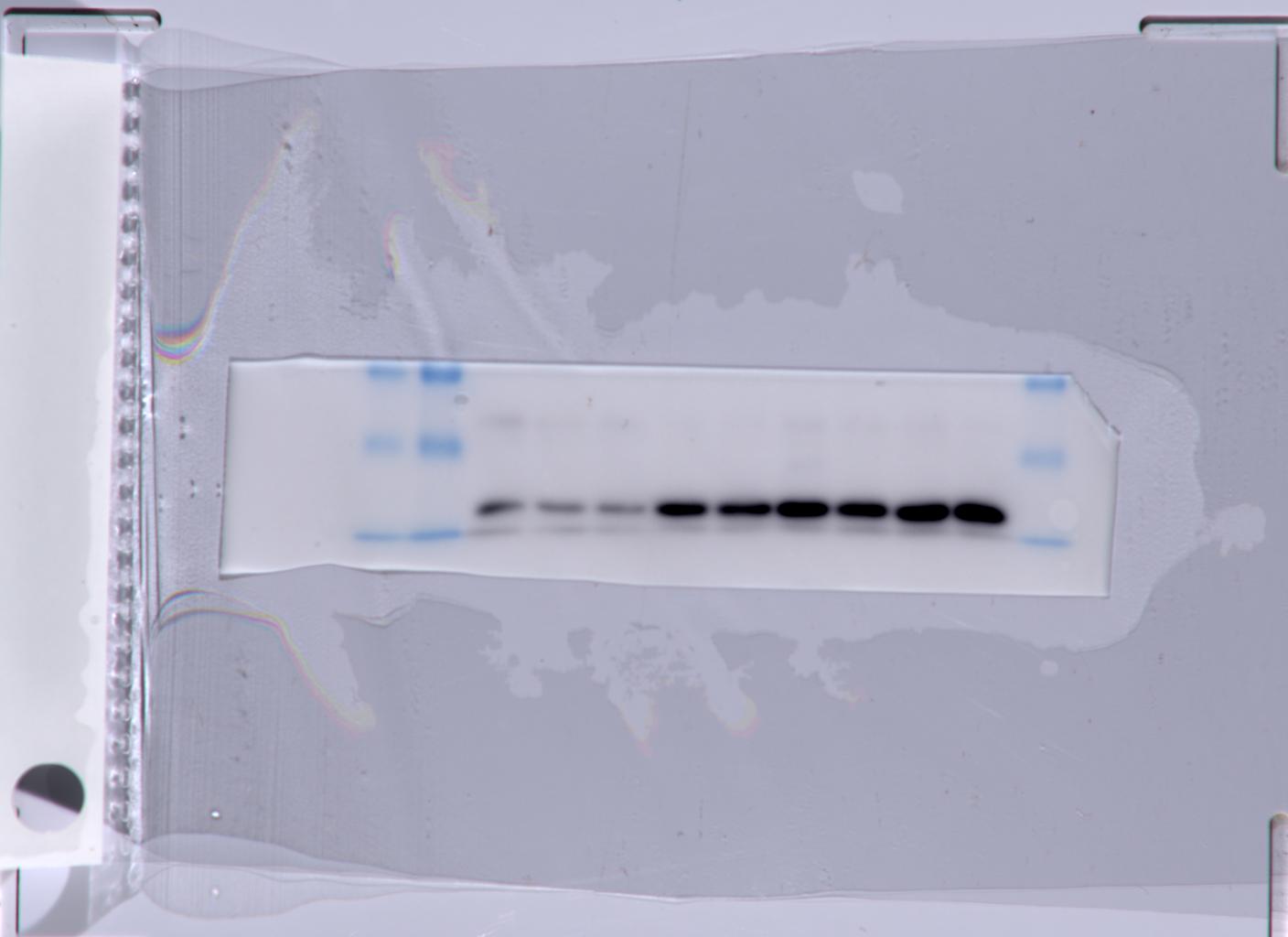

Supplement: Supplementary file 1 [file DataSheet1.zip › Western Raw Data/Figure 5/cre Bax Figure 5.jpeg]

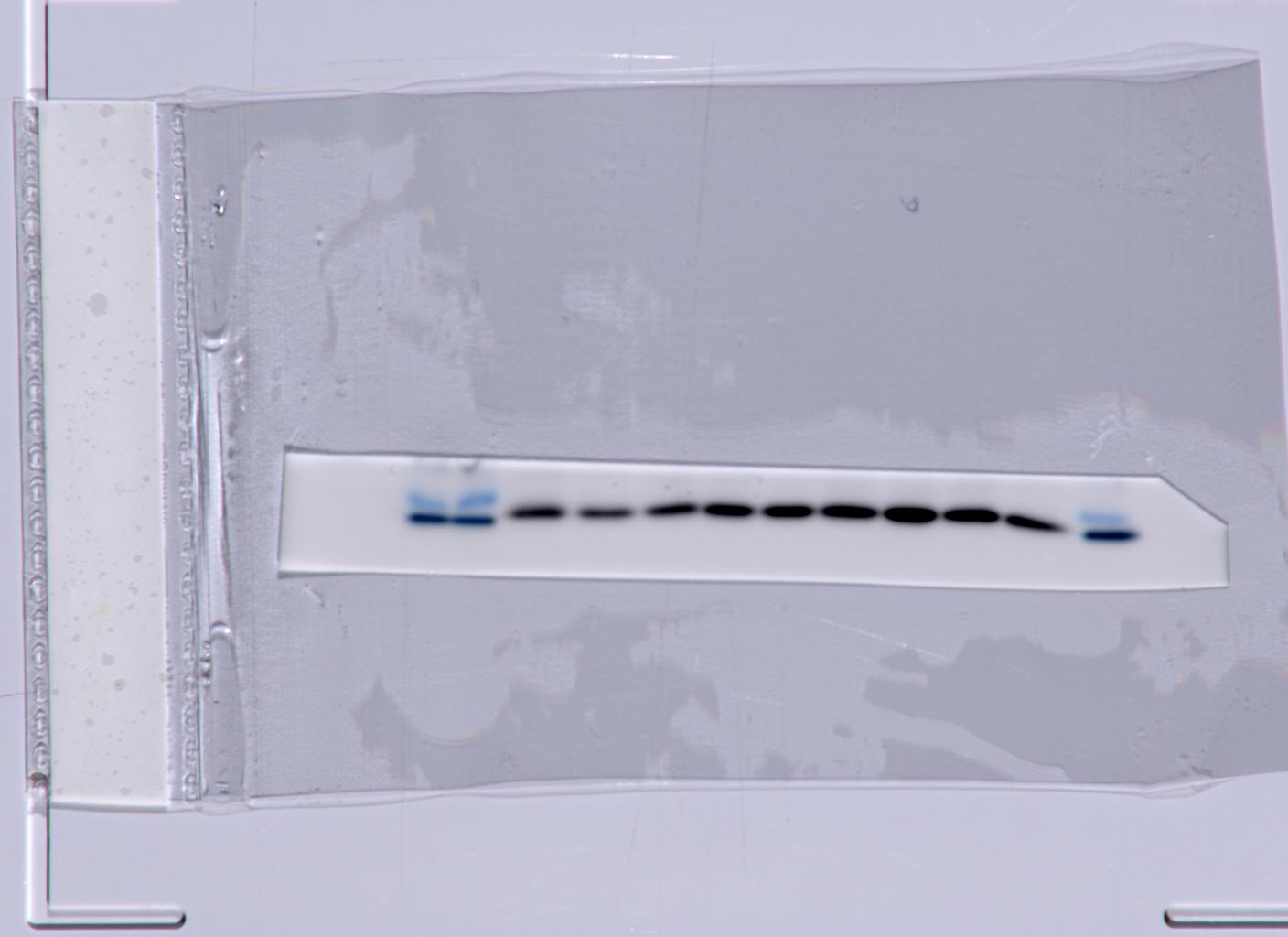

Supplement: Supplementary file 1 [file DataSheet1.zip › Western Raw Data/Figure 5/cre BID Figure 5.jpeg]

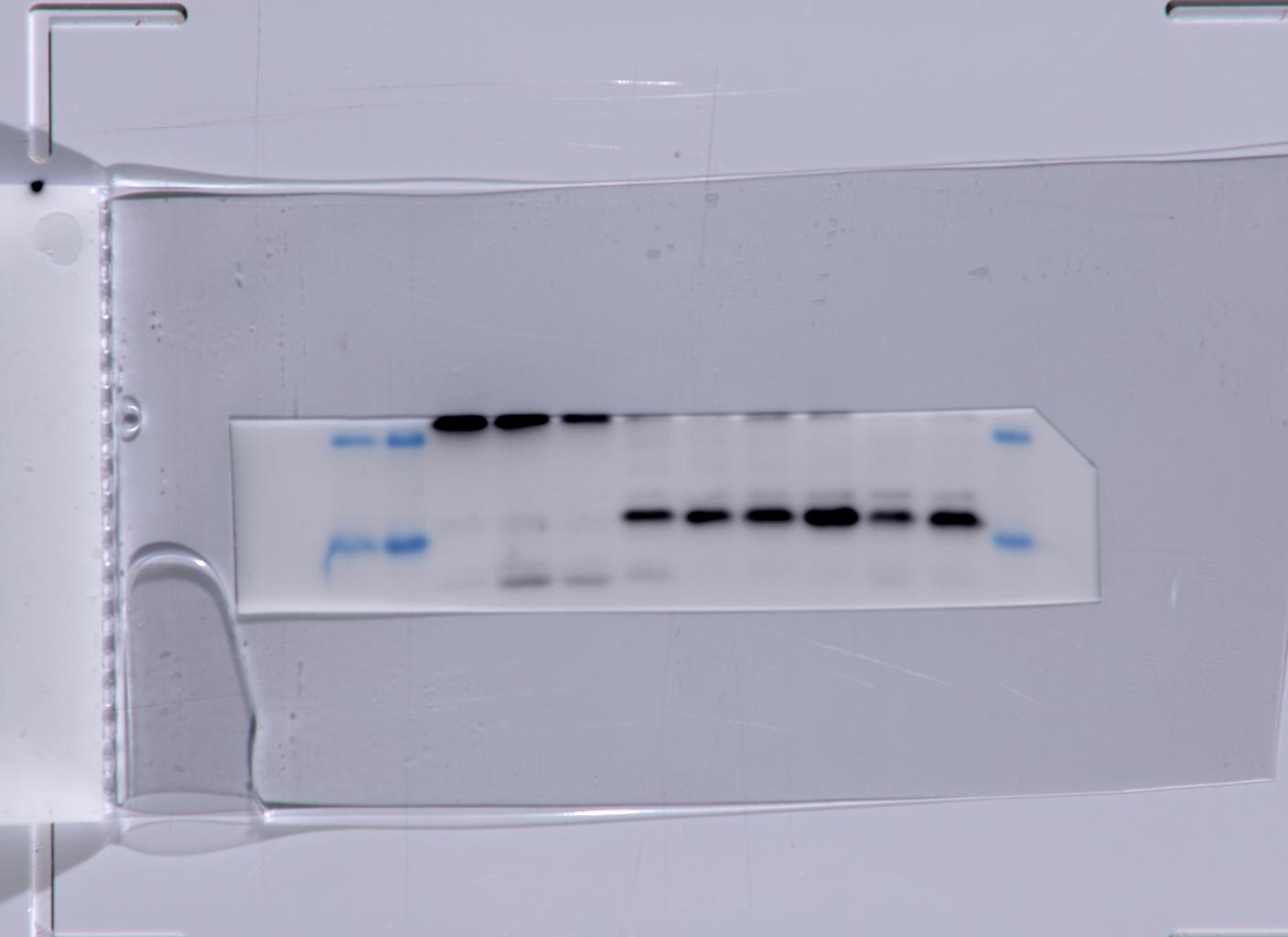

Supplement: Supplementary file 1 [file DataSheet1.zip › Western Raw Data/Figure 5/cre CCas3 Figure 5.jpeg]

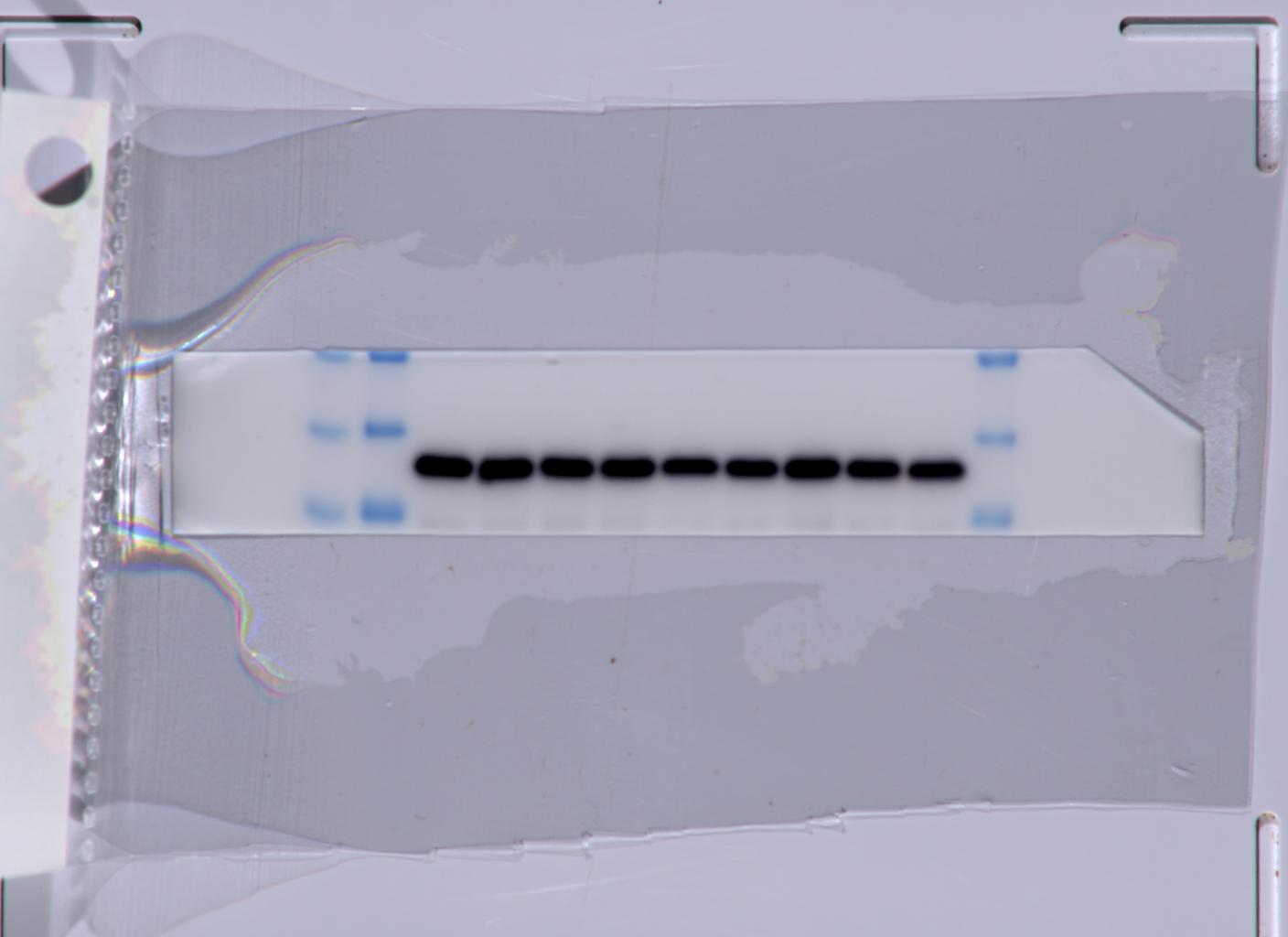

Supplement: Supplementary file 1 [file DataSheet1.zip › Western Raw Data/Figure 5/cre GAPDH Figure 5.jpg]

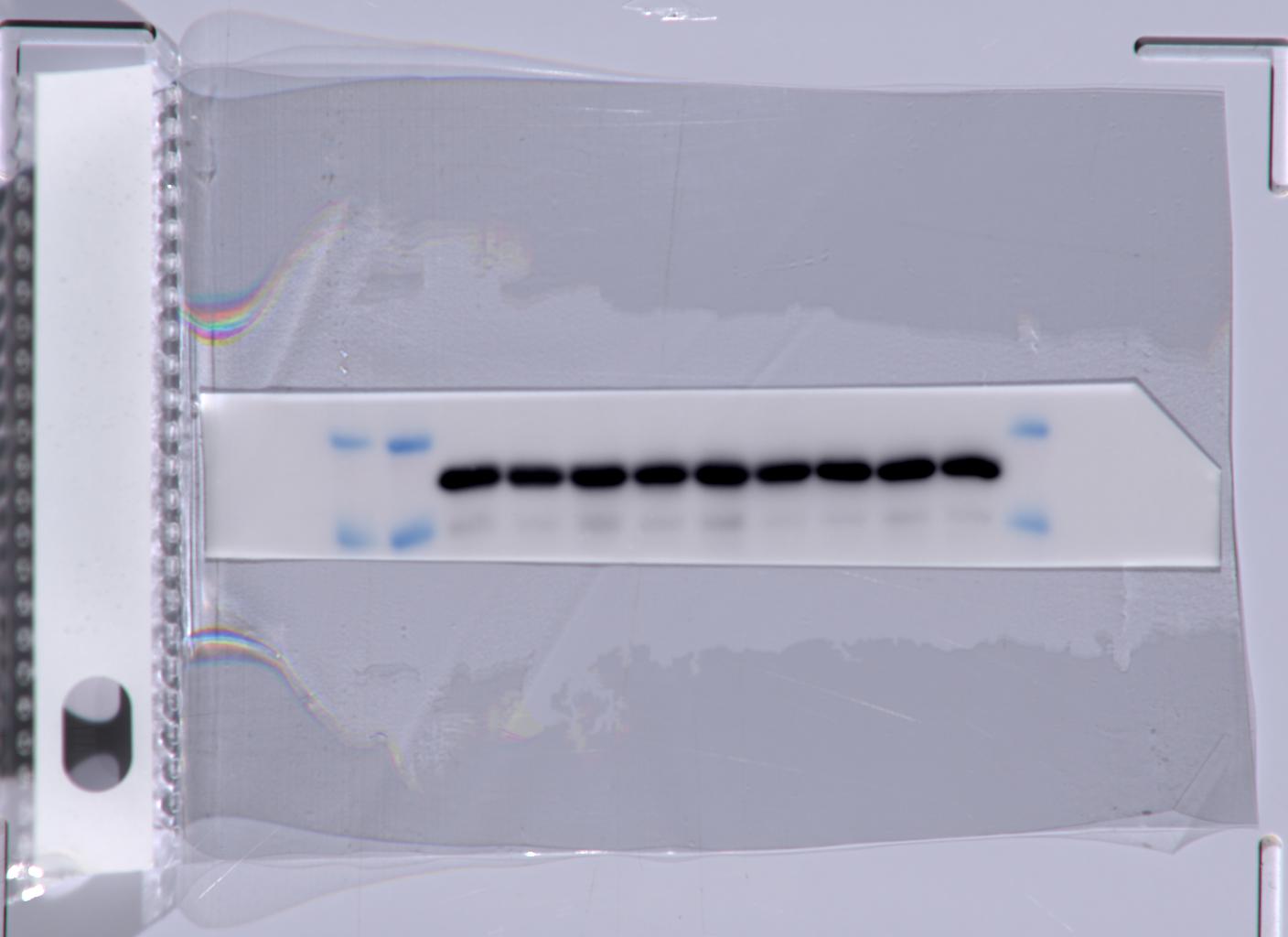

Supplement: Supplementary file 1 [file DataSheet1.zip › Western Raw Data/Figure 5/cre GAPDH Figure 5.jpeg]

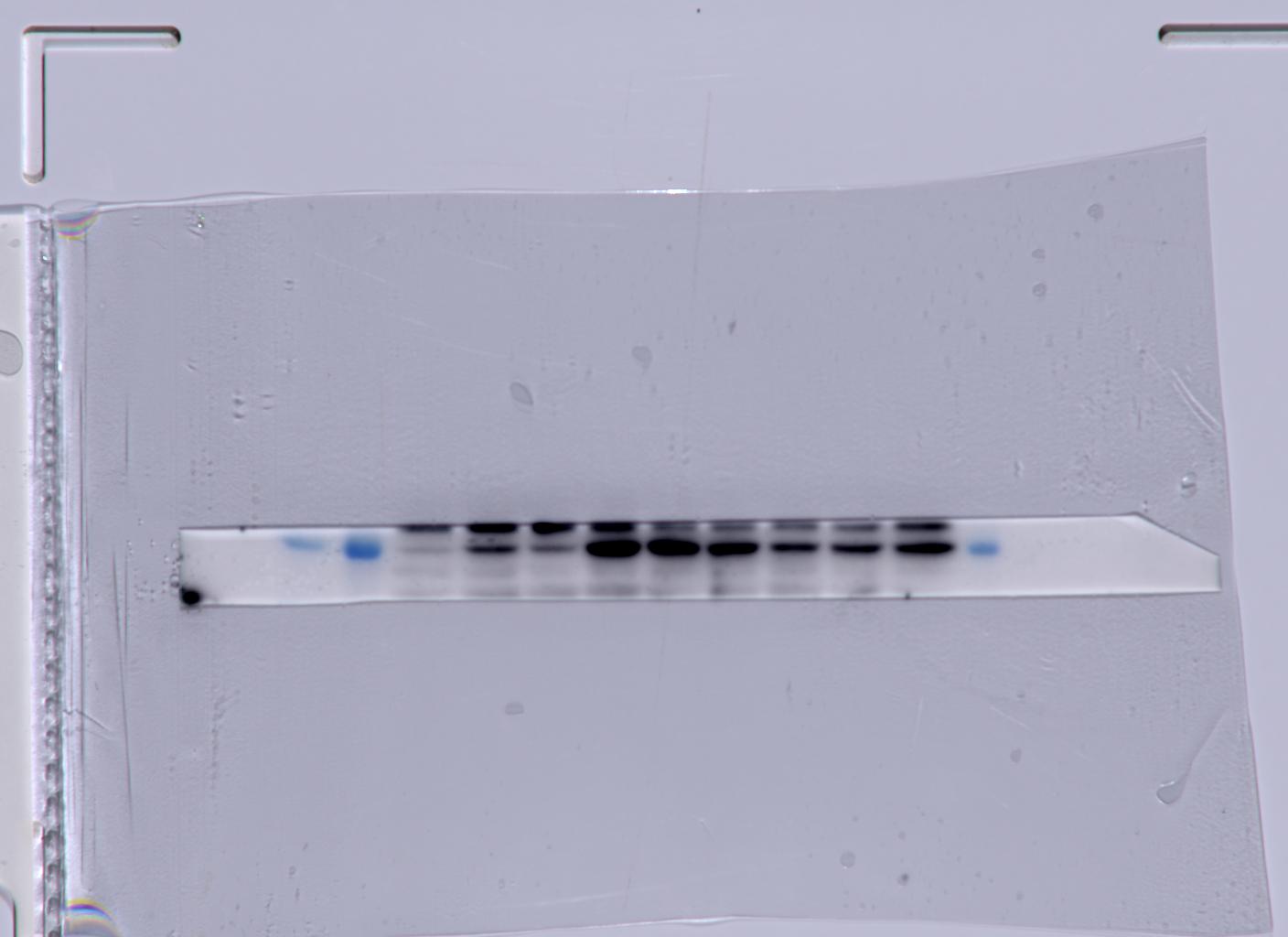

Supplement: Supplementary file 1 [file DataSheet1.zip › Western Raw Data/Figure 5/cre MLKL Figure 5.jpg]

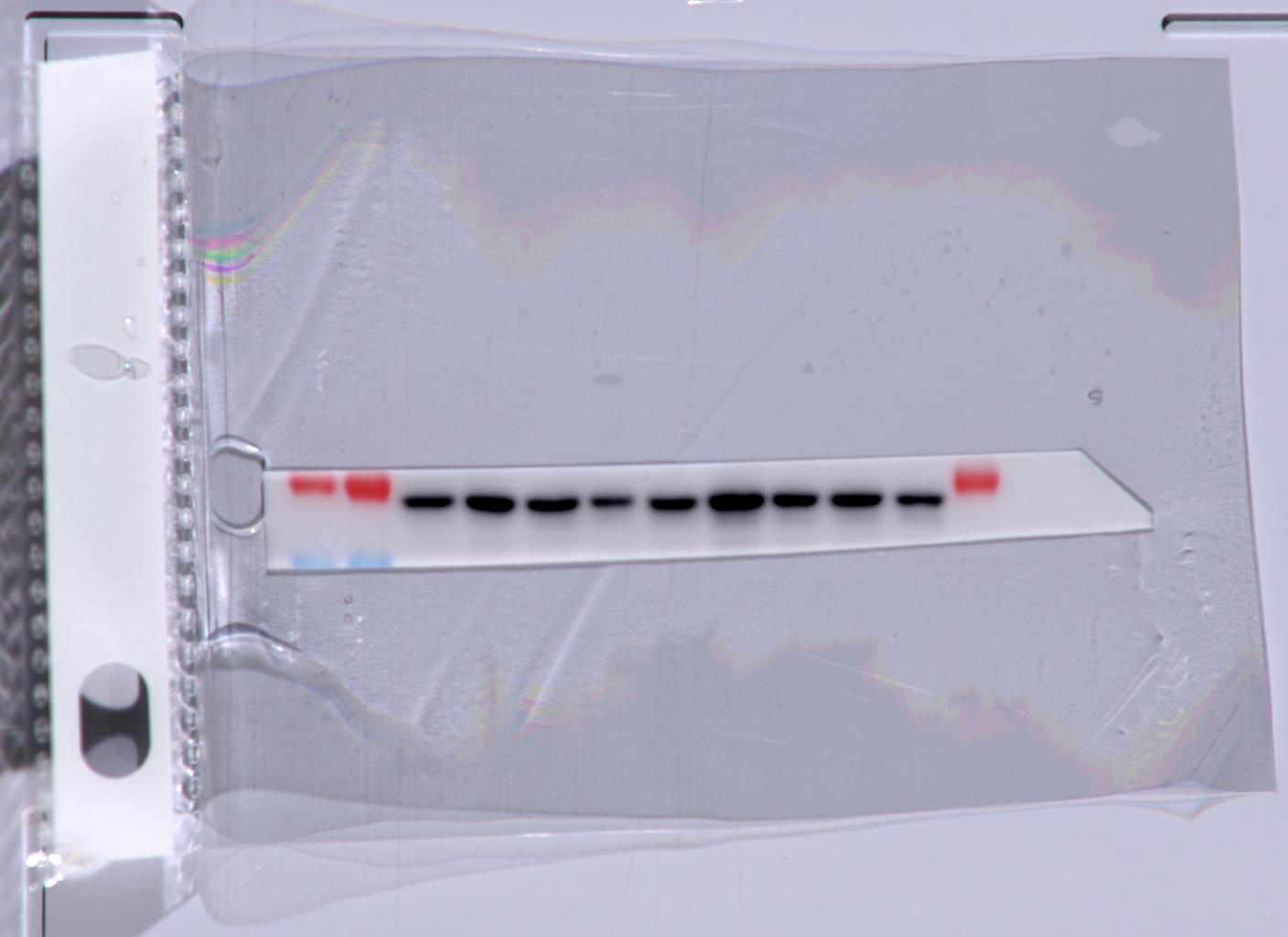

Supplement: Supplementary file 1 [file DataSheet1.zip › Western Raw Data/Figure 5/cre NFkBtot Figure 5.jpeg]

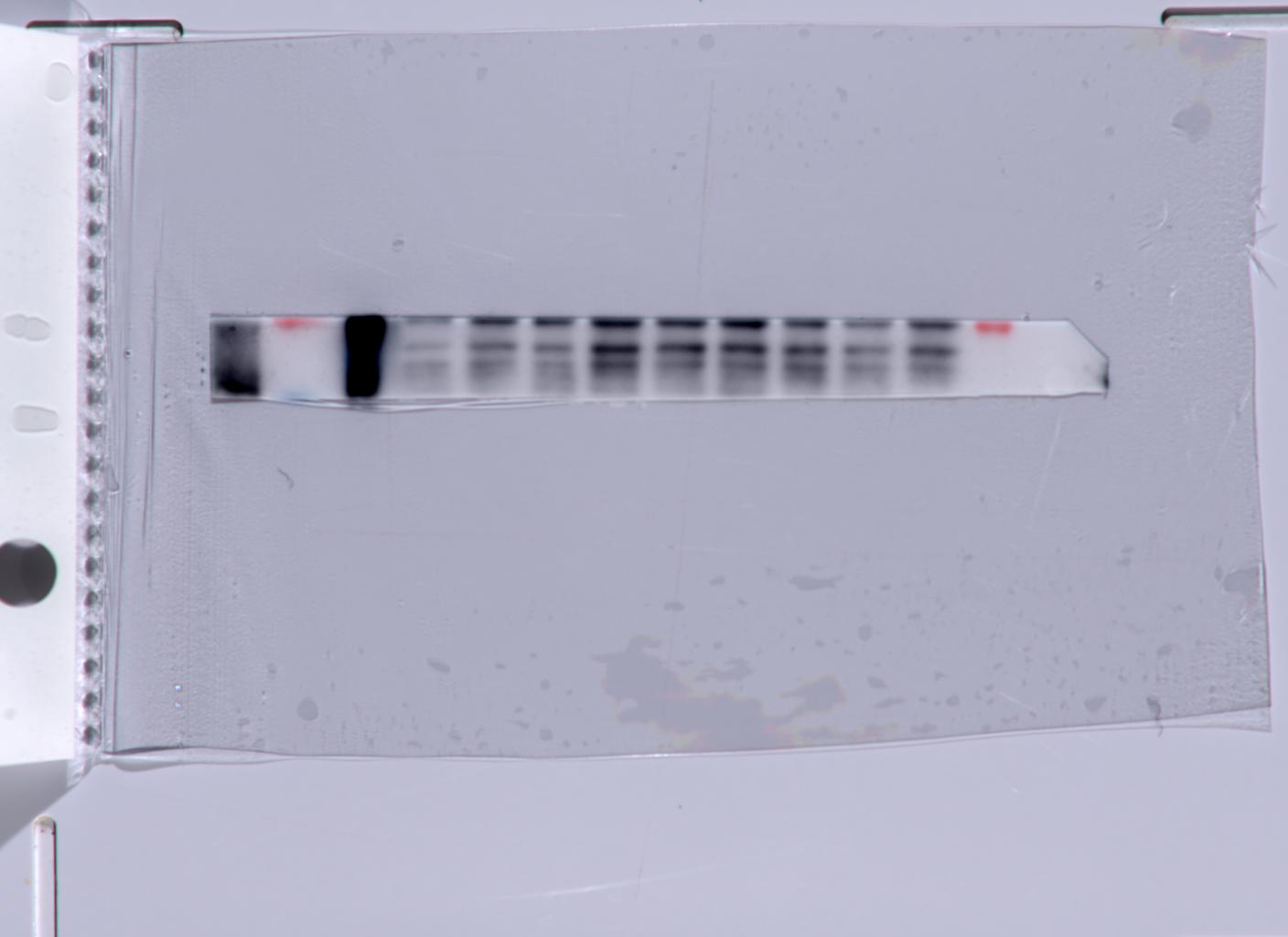

Supplement: Supplementary file 1 [file DataSheet1.zip › Western Raw Data/Figure 5/cre pNFkB ser468 Figure 5.jpg]

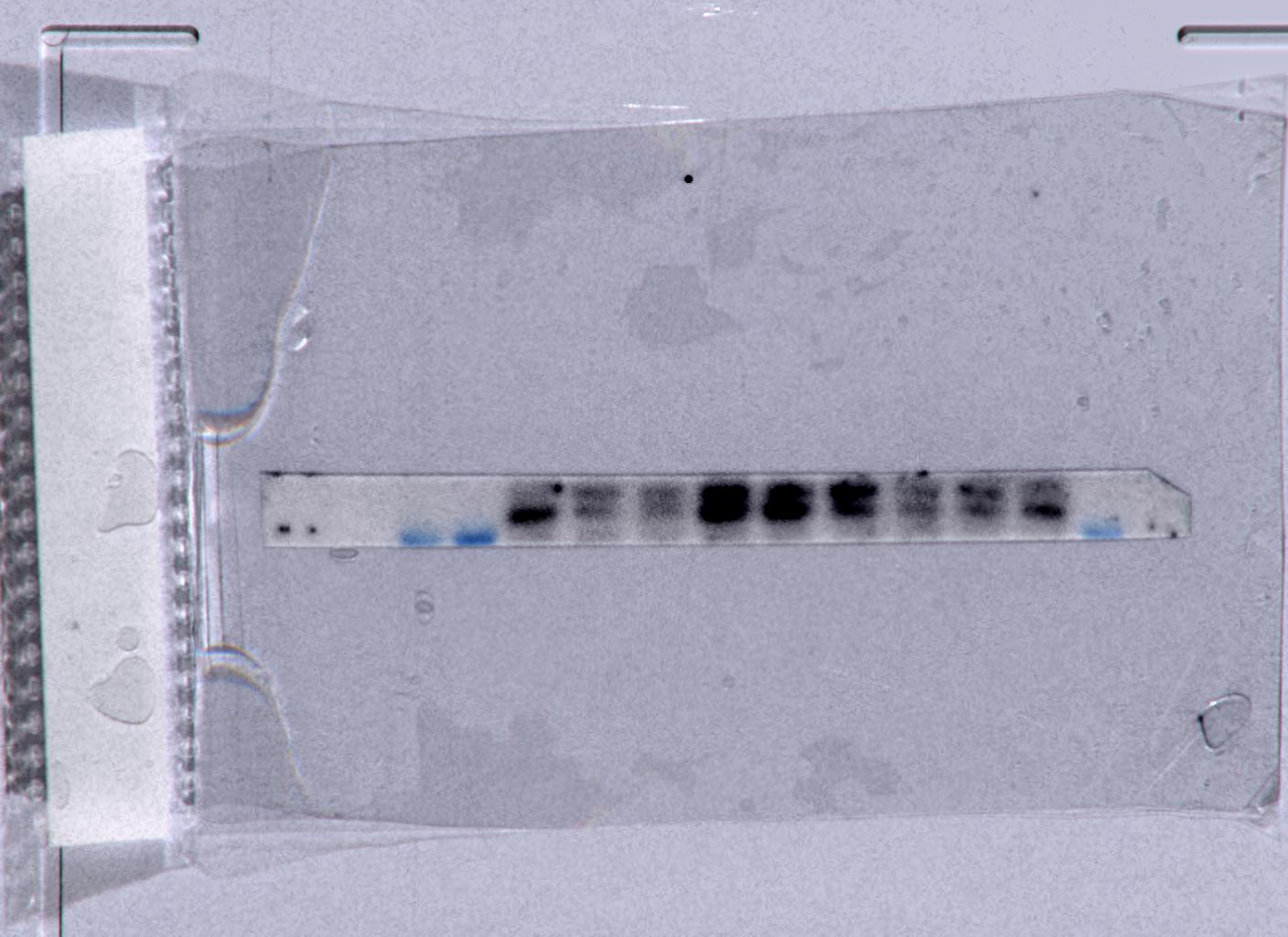

Supplement: Supplementary file 1 [file DataSheet1.zip › Western Raw Data/Figure 5/cre pNFkBS536 Figure 5.jpeg]

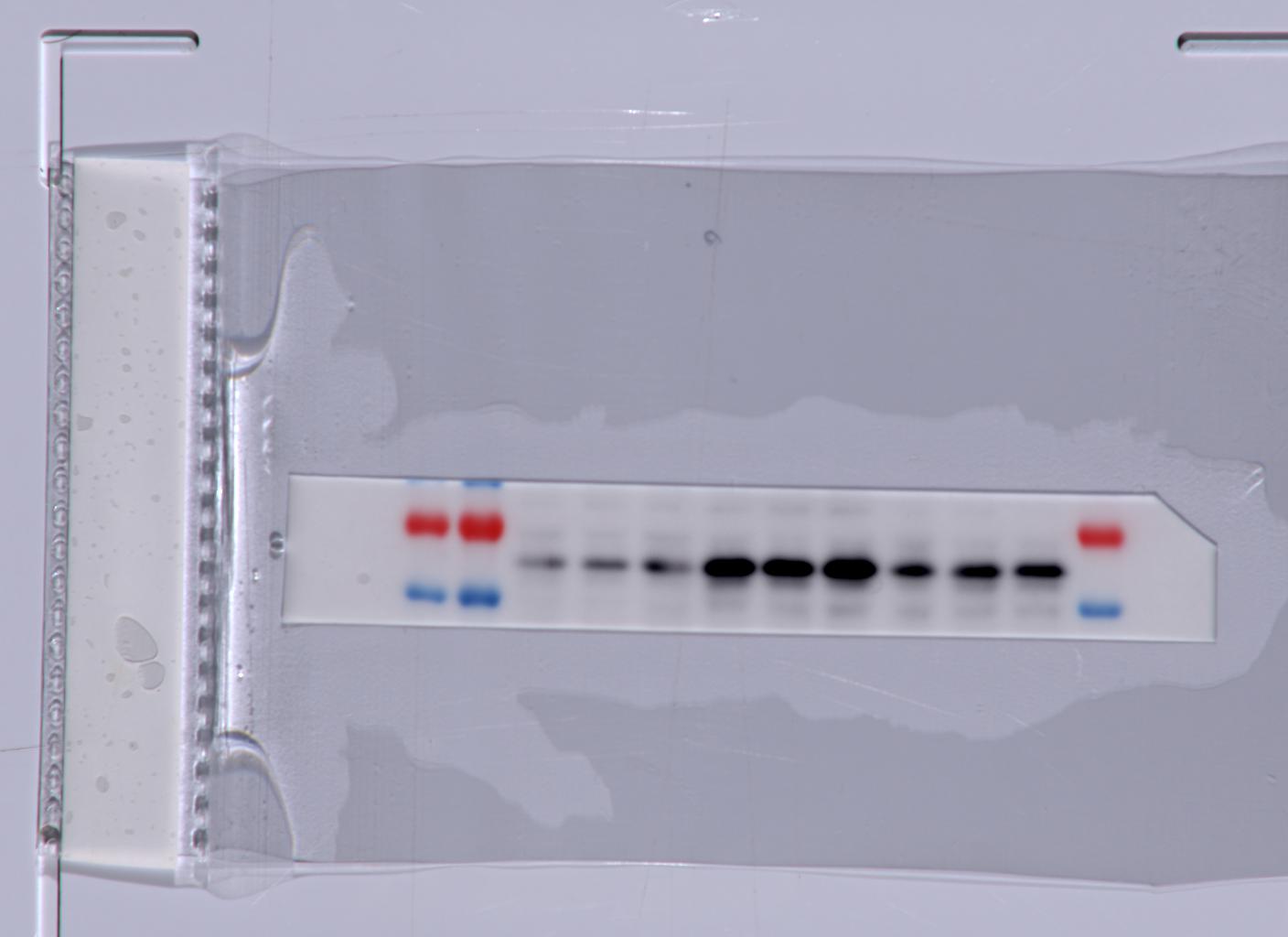

Supplement: Supplementary file 1 [file DataSheet1.zip › Western Raw Data/Figure 5/cre pSMAD2 Figure 5.jpeg]

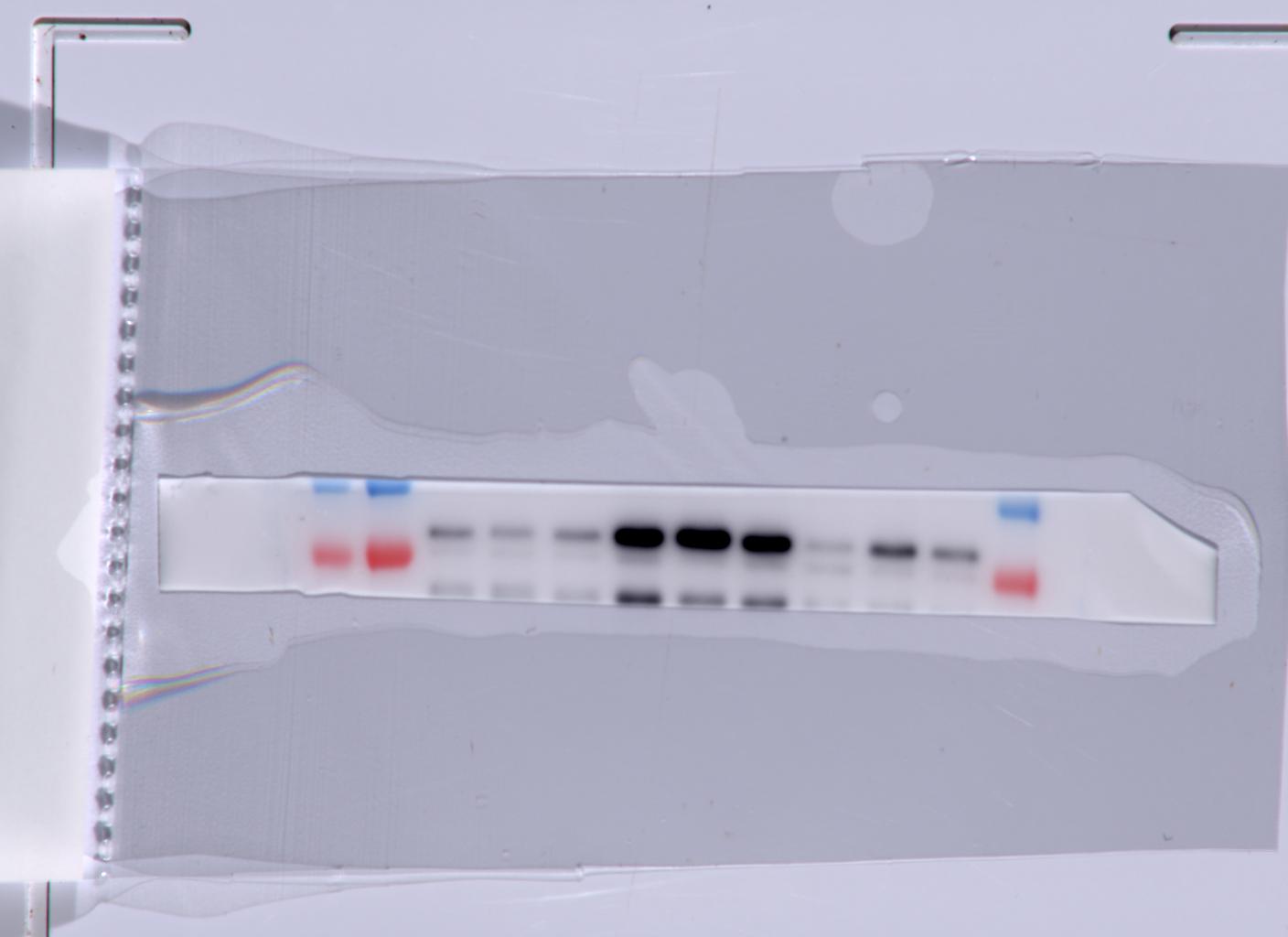

Supplement: Supplementary file 1 [file DataSheet1.zip › Western Raw Data/Figure 5/cre RIPK1 Figure 5.jpg]

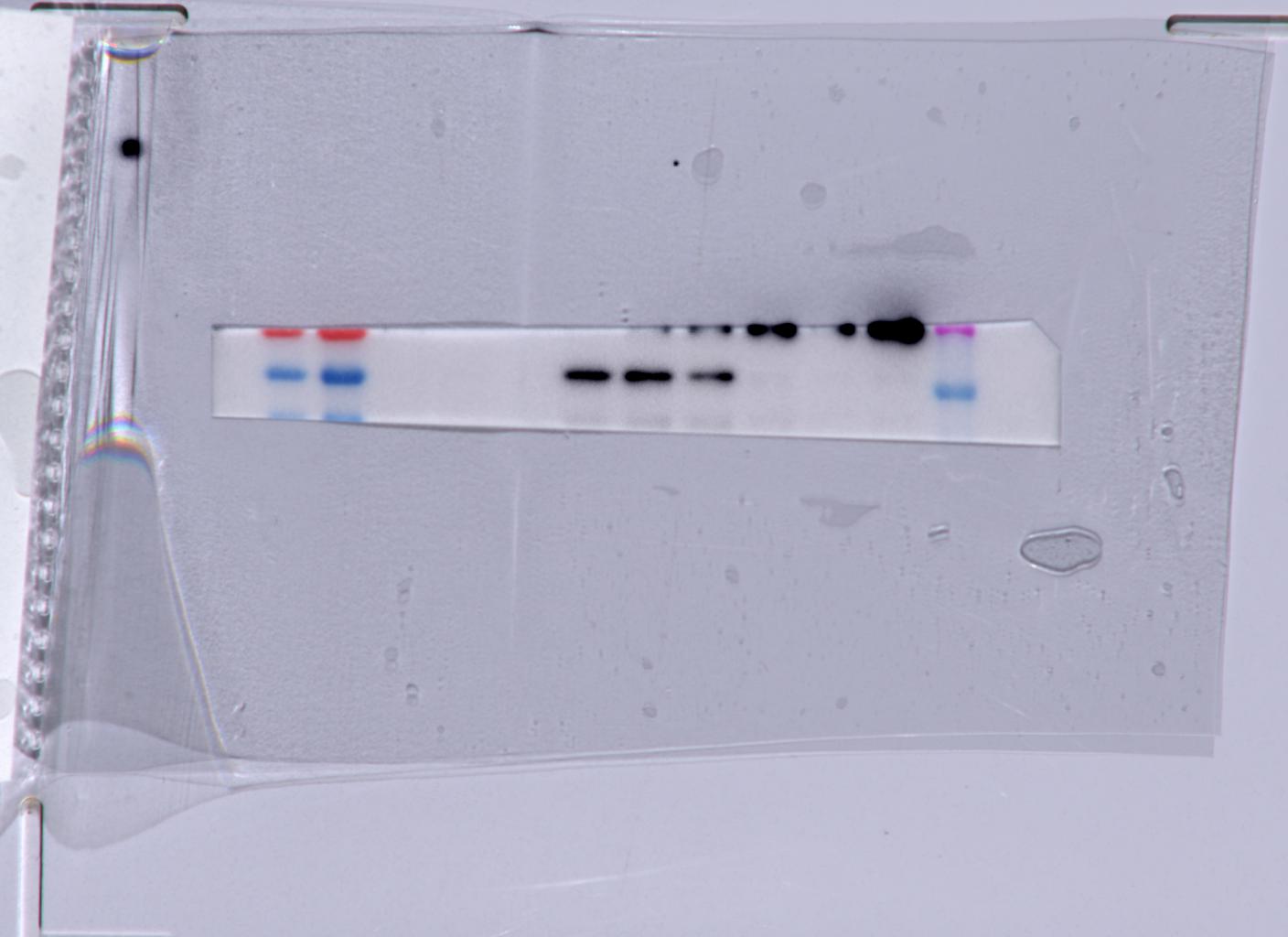

Supplement: Supplementary file 1 [file DataSheet1.zip › Western Raw Data/Figure 5/cre RIPK3 Figure 5.jpg]

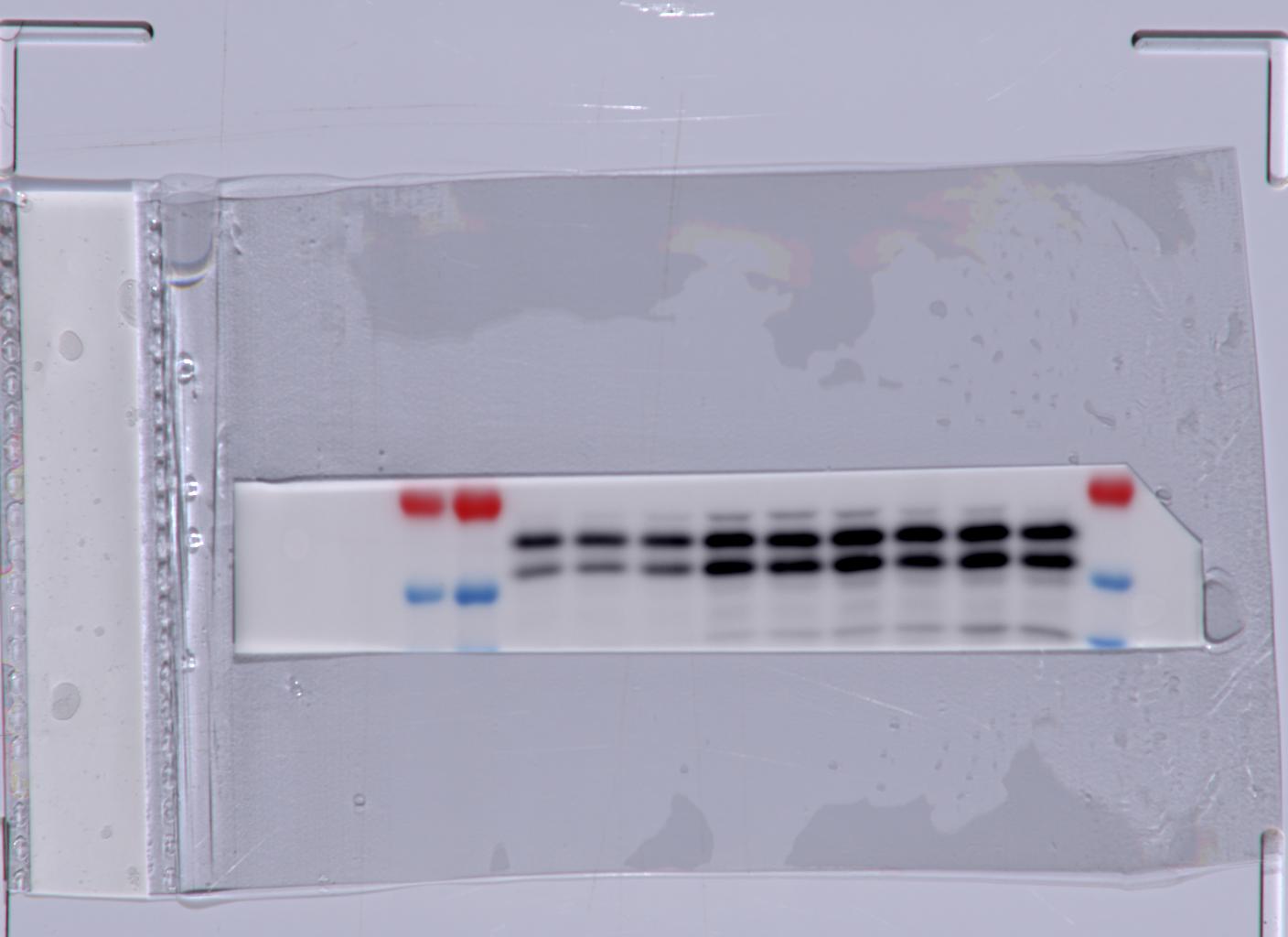

Supplement: Supplementary file 1 [file DataSheet1.zip › Western Raw Data/Figure 5/cre SMAD2and3 Figure 5.jpeg]
